# Characterization of Microbial Dynamics and Volatile Metabolome Changes During Fermentation of Chambourcin Hybrid Grapes From Two Pennsylvania Regions

**DOI:** 10.3389/fmicb.2020.614278

**Published:** 2021-01-11

**Authors:** Hung Li Wang, Helene Hopfer, Darrell W. Cockburn, Josephine Wee

**Affiliations:** ^1^Department of Food Science, The Pennsylvania State University, University Park, PA, United States; ^2^Sensory Evaluation Center, The Pennsylvania State University, State College, PA, United States; ^3^Microbiome Center, HUCK Institute for Life Sciences, The Pennsylvania State University, State College, PA, United States

**Keywords:** wine, hybrid grapes, fermentation, microbiome, metabolome

## Abstract

Microbial diversity present on grapes in wineries, and throughout fermentation has been associated with important metabolites for final wine quality. Although microbiome-metabolome associations have been well characterized and could be used as indicators of wine quality, the impact of regionality on the microbiome and metabolome is not well known. Additionally, studies between microbiome and metabolome have been conducted on single species grape such as *Vitis vinifera* instead of other species and interspecific hybrids. Although the Pennsylvania wine industry is relatively young compared to California, the industry has been experiencing rapid growth over the past decade and is expected to continue to grow in the future. Pennsylvania’s climate of cold winters and high levels of rainfall throughout the growing season favors cultivation of interspecific hybrid grapes such as *Vitis ssp.* Chambourcin, one of the most commonly grown hybrid varieties in the state. Chambourcin is a prime candidate for studying the impact of regionality on microbiome-metabolome interactions as interspecific hybrid varieties could shape the future of winemaking. Here, we identify for the first time the regional distribution of microbial communities and their interactions with volatile metabolome during fermentation (0–20 days) by integrating high throughput Illumina sequencing (16S and ITS) and headspace-solid phase microextraction-gas chromatography-mass spectrometry. Analyzing 88 samples from nine wineries in the Central and East Pennsylvania regions, we observed high microbial diversity during early stages of fermentation (1–4 days) where non-*Saccharomyces* yeasts such as *Starmerella* and *Aureobasidium* and non-*Oenococcus* bacteria, *Sphingomonas*, likely contribute to microbial *terroir* to the resulting wines. Furthermore, key differentiators between two regions in Pennsylvania, as identified by LEfSe analysis, include the fungal genera *Cladosporium* and *Kazachstania* and the bacterial genera *Lactococcus* and *Microbacterium*. Moreover, 29 volatile fermentation metabolites were discriminated significantly (variable importance in projection > 1) between the two regions as shown by Partial Least Squares-Discriminant Analysis. Finally, Spearman’s correlation identified regional differences of microbial-metabolite associations throughout fermentation that could be used for targeted microbiome manipulation to improve wine quality and preserve regionality. In summary, these results demonstrate the microbial signatures during fermentation and differential microorganisms and metabolites further support impact of regionality on Chambourcin wines in Pennsylvania.

## Introduction

Microbial communities play critical roles in complex fermentation systems such as winemaking. Several lines of evidence suggest that changes in microbial diversity and abundance throughout fermentation and winemaking can influence the physicochemical properties of final wines, control wine spoilage, and alter wine perception (Bokulich et al., 2013). In addition, native microbial populations present on grapes, in the vineyard, in the soil, and in wine processing facilities contribute to final wine quality and characteristics relative to sensory properties (Romano et al., 2019). Previous studies have demonstrated that non-*Saccharomyces* genera such as *Hanseniaspora*, *Torulaspora, Pichia*, and *Metschnikowia* can significantly and positively influence flavor profiles of final wine (Pinto et al., 2015; Bozoudi and Tsaltas, 2016; Salvetti et al., 2016; Mezzasalma et al., 2017). For example, co-inoculation of *Pichia kluyveri* with *Saccharomyces cerevisiae* can enhance 3-mercaptohexyl acetate concentration responsible for passion/grape fruit aromas in Sauvignon Blanc wines (Carrau et al., 2020). Additionally, lactic acid bacteria (LAB) such as *Lactobacillus* and *Pediococcus* play important roles in citric acid metabolism and the synthesis of esters such as diethyl succinate esters (fruity aroma) impacting final wine flavor (Inês and Falco, 2018). *Terroir* is a well-known concept to winemakers. *Terroir* is an expression that captures unique features of a region such as environmental factors and winemaking practices that can influence final wines and shape product identity within a wine region (Marlowe and Bauman, 2019). Previous studies have shown that unique microbial populations or “microbial fingerprint” present on grape berries and throughout fermentation associated at a specific geographical location can influence distinct wine characteristics in the wine region (Bokulich et al., 2014; Gilbert et al., 2014). Thus, the contribution of microbial populations on vineyards and in winery environments could also be considered a unique feature contributing *terroir* and can be targeted to enhance final wine quality. Understanding how microbial *terroir* impacts regionality of wine including the characterization of microbial *terroir* related to wine fault and spoilage as well as consumer perception would allow a winemaker to consider practices that preserve and enhance microorganisms within the vineyard and in wineries. This would allow for targeted control or manipulation of microbial *terroir* to increase flavor complexity and preserve regionality (Capozzi et al., 2015; Bokulich et al., 2016).

Pennsylvania (PA) is traditionally known as a large juice and jelly grape producer. The Pennsylvania Wine Industry is emerging as an important economic sector in Pennsylvania and has experienced continuous growth both in terms of number of wineries as well as gallons of wine produced (Thompson et al., 2019). In 2018, sales from PA wineries contributed to approximately $ 418.3 million to the state’s economy (John Dunham and Associates, 2019). One of the major challenges of growing wine grapes in the region is climate. Pennsylvania exhibits cooler temperature with humid climates characteristic of the East Coast of the United States presenting the ideal environment for growth of hybrid grapes compared to *Vitis vinifera* (Reisch et al., 1993; Homich et al., 2016; Thompson et al., 2019). Chambourcin, pronounced “SHAM-bour-sin,” is a French-American hybrid (Seyve-Villard 12-417 x Chancellor) wine grape variety with a relatively dark skin and neutral flavor (Robinson et al., 2012). Compared to *V. vinifera*, previous research suggests that Chambourcin is more tolerant to temperature fluctuations and resistant to cold temperatures (Dombrosky and Gajanan, 2013; Gardner, 2016; Homich et al., 2016). In addition, Chambourcin grape berries are more tolerant to disease pressures such as downy mildew and powdery mildew (Barlass et al., 1987; Hartman and Beale, 2008). In Pennsylvania, this variety is the most abundant hybrid grape grown in the Central, South West, and South East regions, making Chambourcin an important grape cultivar for winemaking (Dewey, 2017). A survey of 39 PA wine and grape growers indicate that winter injury followed by disease pressure is the most relevant challenge in the region (Centinari et al., 2016). Therefore, these versatile and unique characteristics of Chambourcin grown in PA could lead to a more sustainable viticulture resulting in an economic benefit while maintaining wine quality (Santos et al., 2020). Although hybrid grapes represent an important part of many winemaking regions especially in Eastern United States, the majority of wine studies have still focused on *V. vinifera* varieties in warmer climate areas such as California (Homich et al., 2016; Coia and Ward, 2017). Moreover, the wide variety of environmental conditions and viticultural areas within Pennsylvania can contribute to diversity in the microbial *terroir*, potentially leading to distinctive organoleptic wine properties from different regions. To the best of our knowledge, few field studies on actual wineries have explored how microbial diversity and the predominance of unique taxa associates with wine volatiles from hybrid grapes (Bokulich et al., 2016; Mezzasalma et al., 2017). Previous studies of microbial and metabolic dynamics focused on laboratory scale fermentation using selected microbial strains as opposed to relevant industrial environment (Torrea and Ancín, 2002; Azzolini et al., 2013). These gaps in knowledge impedes our understanding of hybrid grape selection for winemaking that could be important when dealing with changing microclimate within wine and grape growing regions. Therefore, investigating the microbiomes associated with hybrid grapes and how this microbial ecology impacts wine aroma characteristics through direct sampling within actual wineries is a necessary first step toward achieving stable and high quality of wines produced by interspecific varieties.

To address this knowledge gap in the impact of microbial populations on hybrid grapes in winemaking, we utilize an Illumina-based next generation sequencing (NGS) approach together with untargeted volatile metabolomics in a Chambourcin model system. Here, our aims are to (1) characterize the Chambourcin fermentation microbiome, (2) determine the impact of regional differences on microbial populations and volatiles, and (3) identify associations between regionally differential microbial taxa and volatile metabolites. To achieve this, we collected 88 commercial samples from 0 to 20 days of fermentation roughly correlating with early, mid-, and late fermentation stages of winemaking from wineries in the Central and East regions of Pennsylvania. To characterize wine volatile metabolites, we used gas chromatography-mass spectrometry (GC-MS) with headspace-solid-phase microextraction (HS-SPME) for non-targeted metabolite profiling of volatile compounds in all samples. Together, our work provides important insights into distinct regional characteristics of microbiome dynamics and volatile metabolome during the Chambourcin fermentation process. Therefore, understanding regional microbial signatures and volatile metabolites would allow for future targeted microbiome manipulation to improve Pennsylvania wine characteristics and competitiveness on a national market.

## Materials and Methods

### Sample Collection and DNA Extraction

Samples were collected during the 2019 vintage from a total of 9 commercial wineries located in two PA regions, the East region (*n* = 4) and the Central region (*n* = 5; Supplementary Figure 1). Wineries were located within ∼ 36 km in the East region and within 73 km in the Central region, with up to 228 km between wineries from the two regions. All selected wineries and vineyards grew and processed their own Chambourcin into wine. Participating wineries were provided with sample collection and handling instructions which included a survey log for sample handling outlining fermentation stages designated S1–10 based on time. These pre-determined fermentation stages for sample collection were selected based on recommendations by head winemakers from two of the nine participating wineries taking into account practical aspects of sampling. Of particular importance in this study, wineries did not change existing winemaking procedures, and thus were not prevented from using commercial *S. cerevisiae* for initial fermentation and/or *O. oeni* to initiate malolactic fermentation (MLF), however, this information was asked for in the survey log (Supplementary Table 1). Preserving winemaking practices in this study was important to capture individual winery practices that may influence microbial communities and volatile metabolite compositions. At each of the 10 pre-determined sampling points, 50 mL of the fermenting must/wine were collected in duplicate into provided sterile centrifuge tubes (VWR, Radnor, PA, United States) over a 20-day period (Supplementary Table 2); the sampling protocol was developed together with two winemakers at two of the participating wineries. Samples were stored immediately after sampling at −20°C until pick-up by the research team, transfer on dry ice to the Penn State campus at University Park, PA within 1 day, and further storage at −80°C until microbiome and metabolome analyses. Due to uncontrollable circumstances, one sample was lost during transportation and another sample was not collected during winemaking by winery staff resulting in a total of 88 unique samples, sampled in biological duplicate (*n* = 88; 9 wineries × 10 fermentation stages minus 2 incomplete samples that were unable to process due to transport and handling issues).

Total genomic DNA was extracted and prepared for microbiome sequencing as previously published with minor modifications (Bokulich et al., 2016). Samples from different fermentation stages and wineries were thawed and centrifuged at 8,000 × *g* for 15 min and supernatants were discarded. Next, pellets were washed with ice-cold phosphate-buffered saline, PBS (pH 7.4) prepared based on the protocol (Cold Spring Harbour Laboratories, 2006) and centrifuged at 8,000 × *g* for 15 min. Wash and centrifugation steps were repeated three times. DNA was extracted from approximately 200 mg of washed pellets from each sample using Quick-DNA^TM^ Fecal/Soil Microbe Miniprep DNA extraction kit (Zymo Research, Irvine, CA, United States). DNA concentration obtained of each sample was quantified using NanodropOne (Thermo Scientific, Waltham, MA, United States) and quality was monitored by the 260/280 ratio. DNA samples were normalized to 3 ng/μL by dilution with nuclease-free water (Life Technologies Corporation, Carlsbad, CA, United States) and stored at −80°C until further use.

### Library Preparation and Sequencing

Fungal and bacterial populations in collected samples were characterized by amplicon-based sequencing of the internal transcribed spacer 2 (ITS2) sequence and the V4 domain of 16s rRNA gene, respectively. The first round PCR amplification (25 μL reaction volume) for each sample included 12 ng of DNA template, 1 × KAPA HiFi HotStart ReadyMix (Kapa Biosystems, Wilmington, MA, United States), 0.25 μM of each primer, nuclease-free water (Life Technologies Corporation, Carlsbad, CA, United States), and 0.05 mg/mL bovine serum albumin (BSA; Sigma-Aldrich, Saint Louis, MO, United States). The fungal ITS2 locus was amplified using the forward primer ITS9 (5′-GAA CGC AGC RAA IIG YGA-3′) and reverse primer ITS4 (5′-TCC TCC GCT TAT TGA TAT GC-3′; Nordberg et al., 2014), with forward Illumina adapter overhang sequences (5′-TCG TCG GCA GCG TCA GAT GTG TAT AAG AGA CAG-[ITS2 sequences]-3′), and reverse Illumina adapter overhang sequences (5′- GTC TCG TGG GCT CGG AGA TGT GTA TAA GAG ACA G-[ITS2 sequences]-3′; PCR Amplicon, PCR Clean-up, and Index PCR, 2013). PCR amplification was carried out initially at 98°C for 5 min, followed by 30 cycles at 95°C for 45 s, 55°C for 60 s, and 72°C for 60 s, and a final extension at 72°C for 5 min. The V4 region of bacterial 16S rRNA genes were amplified with forward primer 515F (5′-GTG YCA GCM GCC GCG GTA A-3′; Parada et al., 2016) and reverse primer 806R (5′-GGACTACNvGGGTWTCTAAT-3′; Apprill et al., 2015), with forward Illumina adapter overhang sequences (5′-TCG TCG GCA GCG TCA GAT GTG TAT AAG AGA CAG-[16S rRNA v4 genes sequences]-3′), and reverse Illumina adapter overhang sequences (5′- GTC TCG TGG GCT CGG AGA TGT GTA TAA GAG ACA G-[16S rRNA v4 genes sequences]-3′). Reaction conditions consisted of 98°C for 2 min, followed by 25 cycles at 95°C for 15 s, 59°C for 15 s, and 72°C for 15 s, with a final extension at 72°C for 5 min. PCR amplicons were purified using GenElute^TM^ PCR Clean-Up Kit (Sigma-Aldrich, Saint Louis, MO, United States) to remove single primers and primer dimers.

Purified PCR amplicons were submitted to The Pennsylvania State University HUCK Institutes of the Life Sciences Genomics Core Facility for Illumina paired-end library preparation, cluster generation, and 250-bp paired-end sequencing. Purified fungal and bacterial PCR amplicons from every twelve samples were pooled together and analyzed using a 2100 Bioanalyzer (Agilent Technologies, Inc., Santa Clara, CA, United States) to assess untargeted artifacts present in samples for quality control. The same quality control and cleanup protocol was applied to the index PCR step. Equimolar concentrations of pooled libraries containing PCR amplicons were sequenced using 250-bp paired-end sequencing on an Illumina MiSeq instrument (Illumina, San Diego, CA, United States).

### Microbiome-Based Bioinformatic Data Analysis Pipeline

Raw sequences obtained from Illumina MiSeq comprising of bacterial and fungal DNA were analyzed using QIIME2 v2019.7 (Bokulich et al., 2018) and the resulting data in Casava 1.8 paired-end demultiplexed format was imported using the qiime tools import plugin.

Forward and reverse reads of bacterial 16S rRNA gene sequences were truncated at base position 196 and 204, respectively, followed by denoising with the q2-DADA2 plugin (Callahan et al., 2016). One sample (PA19_02_S10) was first removed due to low sequencing reads (reads < 1,000). Amplicon sequence variants (ASVs) from a total of 86 bacterial samples were classified using the q2-feature-classifier plugin and a pre-trained Naïve Bayes classifier (Bokulich et al., 2018) with the SILVA 128 99% OTU reference database (Quast et al., 2013) for taxonomic identification. Another sample (PA19_09_S4) was removed due to unidentified taxonomic sequences. To obtain a phylogenetic tree for diversity analyses, we used a fragment-insertion plugin based on the SEPP algorithm (Janssen et al., 2018) to phylogenetically place the ASVs into the high quality preconstructed reference SILVA v128 99% identity tree (Yilmaz et al., 2014).

Raw fungal ITS2 sequences were trimmed using the q2-ITSxpress plugin (Rivers et al., 2018) and denoised using the q2-DADA2 plugin (Callahan et al., 2016). Three samples were removed due to low sequencing reads (reads < 3,000), so a total of 85 fungal samples were used for the following procedures. The q2-feature-classifier and a Naïve Bayes classifier were used for fungal taxonomy identification with the UNITE ver8 99% OTU (UNITE Community, 2019) database, trained on the full reference sequences without any extraction. To obtain a phylogenetic tree for diversity analyses, fungal ASVs were pre-filtered if sequences were lower than 80% identity to any reference sequence and clustered against the UNITE ver8 99% OTUs reference database (UNITE Community, 2019), using the QIIME vsearch cluster-features-closed-reference plugin (Nilsson et al., 2019). Clustered sequences were then aligned with a pre-built phylogenic reference tree made by the UNITE ITS extension database and the SILVA 18S database using the q2-ghost-tree plugin to construct a reference-based fungal phylogenetic tree (Fouquier et al., 2016).

The relative abundance of non-*Saccharomyces* or non-*Oenococcus* taxa at the genus level was normalized using the values determined by the reads per taxon divided by the number of summing reads for each sample (reads of *S. cerevisiae* or *O. oeni* were excluded). The relative abundance of *S. cerevisiae* and *O. oeni* was determined by the reads of the taxon at the species level divided by the number of summing reads for each sample. Alpha-diversity (within-sample) was measured using Faith’s phylogenetic diversity (Faith’s PD; Faith, 1992) and Pielou’s Evenness (Pielou, 1966) within the q2-diversity plugin using rarefied counts (i.e., normalized to the same reads across samples; normalized to 1,669 for the 16S dataset, and 16,306 for the filtered ITS2 dataset). Boxplots for alpha diversity were created using the R package ggplot2 (Wickham, 2016) in R version 3.6.3 (R Core Team, 2020) via RStudio version 1.2.1335^1^. Pairwise comparisons between fermentation stages relative to stage 1 were performed using the Kruskal–Wallis rank-based approach for non-parametric data. A false discovery rate (FDR) adjusted *p*-value (*q*-value) was used to indicate statistical significance (*q*-value < 0.05; Nahm, 2016). Beta diversity was performed for quantitative measures of microbial community dissimilarity using weighted UniFrac distance metrics (Lozupone et al., 2007). Distance metrics were exported from QIIME2 and imported into R to be visualized in a Principal Coordinate Analysis (PCoA) plot using the R package qiime2R (Bisanz, 2018). Pairwise comparisons of beta diversity were tested using permutational multivariate analysis of variance (PERMANOVA) with 999 permutations, a non-parametric approach of multivariate analysis of dissimilarity based on pairwise distances (McArdle and Anderson, 2001; Supplementary Table 3).

Differentiation of microbial communities between Central and East regions at the different taxonomic levels was analyzed by the Linear discriminant analysis (LDA) Effect Size (LEfSe). Any fungal or bacterial taxa representing less than 0.01% of the total bacterial or fungal reads was filtered to avoid the influence of erroneous reads. LEfSe supports multidimensional groups comparisons and enables identification of differences between groups by coupling standard tests for statistical significance; here, a non-parametric factorial Kruskal–Wallis (KW) sum-rank test and LDA scores were used to estimate the effect size of differentially abundant taxa (Segata et al., 2011). Microbial communities were considered significantly different if their differentiation between two regions had a *p*-value < 0.05 and a log10 transformed LDA score > 3.

### Volatile Compounds Analysis

Volatile compounds present in the 88 samples obtained at each of the 10 fermentation stages were analyzed by headspace solid phase microextraction coupled to gas chromatography-mass spectrometry (HS-SPME-GC-MS; Agilent Technologies, Santa Clara, CA, United States), using a Robotic autosampler (Gerstel, Linthicum Heights, MD, United States). A 2 cm 50/30 μm divinylbenzene- carboxen-polydimethysiloxane (DVB/CAR/PDMS) SPME fiber (Sigma-Aldrich, Inc., Saint Louis, MO, United States) was chosen for extracting volatile compounds, based on previous literature (Hopfer et al., 2013). Each sample vial contained 2 mL of sample, 3 *g* of NaCl (DotScientific, Burton, MI, United States), 0.5 *g* D-gluconic acid lactone (Sigma-Aldrich) as an inhibitor of grape β-glucosidase activity (Pedneault et al., 2013) and 10 μL of an internal standard (IS; 2-octanol 13.7 mg/L and d8-naphthalene 9.9 mg/L in Methanol; Sigma-Aldrich) for the normalization of volatile compounds from each sample. Each vial was incubated at 30°C for 5 min with shaking at 250 rpm after which the SPME fiber was exposed to the headspace for 30 min at 30°C. Extracted volatiles where thermally desorbed for 10 min in the hot (250°C) inlet equipped with a SPME inlet liner (Sigma-Aldrich) and separated in constant flow mode (1 mL/min ultrapure Helium, Praxair, State College, PA, United States) on a Rtx-WAX capillary column (30 m × 0.25 mm ID, 0.25 μm film thickness; Restek, Bellefonte, PA, United States) with an oven program starting at 30°C for 1 min, followed by a 10°C/min temperature ramp to 250°C, with a final hold of 5 min. Volatiles were identified in scan mode (33–350 amu; 8 scans/s) in electron ionization (EI) at 70 eV with the MS interface, ion source and quadrupole temperatures held at 250, 230, and 150°C. An alkane standard (C8-C20; Sigma-Aldrich) was analyzed alongside the samples to calculate retention indices (RIs) for each metabolite. Data from a total of 83 samples were further processed described below as 5 samples (PA19_05 S1-S5) were lost during laboratory preparation.

For GCMS data processing, common contaminating ions (147, 148, and 149, 207, 221, 267, and 281 m/z) were removed, followed by the Savizky-Golay filter in OpenChrom version 1.3.0 (Wenig and Odermatt, 2010). The PaRAllel FACtor analysis 2 (PARAFAC2)-based Deconvolution and Identification System (PARADISe) version 3.9 (Johnsen et al., 2017) was used to deconvolute overlapping signals, lower the signal-to-noise (S/N) ratio of chromatographic peaks, and address retention time shifts. The settings used were non-negativity and performance of 5,000 iterations for manual set retention time intervals. One to seven components calculated from the model were determined by the user to differentiate the underlying co-eluting metabolites and baseline. Deconvoluted mass spectra were then identified using the National Institute of Standards and Technology (NIST14) mass spectral library version 2.2 (Mikaia et al., 2014). A spectral library match of at least 70%, a verification of calculated RIs with literature values, and the compound being present at the last fermentation stage (S10) in all nine wineries were set as the selective cutoff for identified metabolites (*n* = 64; Supplementary Table 4). The rationale for selection of these core Chambourcin metabolites was that metabolites identified in the final fermentation stage (S10; day 20) more closely mimic final Chambourcin wine composition and thus represent important Chambourcin wine metabolites. For statistical analysis, volatile metabolites were log2-transformed to correct for data skewness and Pareto-scaled to reduce the effect of highly abundant metabolites, using MetaboAnalyst version 4.0 (Chong et al., 2019), prior to partial least squares-discriminant analysis (PLS-DA).

Partial least squares-discriminant analysis was performed in MetaboAnalyst to identify volatiles that differed between the two regions throughout fermentation. Volatiles with variable importance in projection (VIP) values of greater 1 were deemed important for the differentiation of regional volatile profiles. Loading values from principle component 1 and 2 were listed for the importance of volatile compounds in the PLS-DA model (Supplementary Table 5). The quality of the PLS-DA model was estimated by the cumulative *R*^2^, representing the coefficient of determination (goodness of fit), and the cumulative *Q*^2^, representing the coefficient of prediction (goodness of prediction), calculated by Leave-one-out cross-validation (LOOCV; Supplementary Figure 2). To test the effectiveness of the PLS-DA discrimination model, a permutation test with 1,000 permutations was conducted based on the ratio of between group sum of squares and within group sum of squares (B/W-ratio; Supplementary Figure 3).

### Correlation Analyses of Microbiome and Metabolomes

A list of significantly different fungal and bacterial populations from LEfSe analysis and VIP scores of volatile metabolites from PLS-DA were extracted from the Central and East regions. To visualize associations between fungal or bacterial taxa and volatile metabolites, a Spearman’s rank correlation was performed using the ggcorrplot R package where correlation coefficients with FDR-adjusted *p*-value of less than 0.05 were retained in the correlation heatmaps (Alboukadel Kassambara, 2019).

## Results

### Microbial Diversity During Early Stages of Chambourcin Fermentation Provides Insights Into Pennsylvania Regional Identity of Microbiome

**FIGURE 1:**
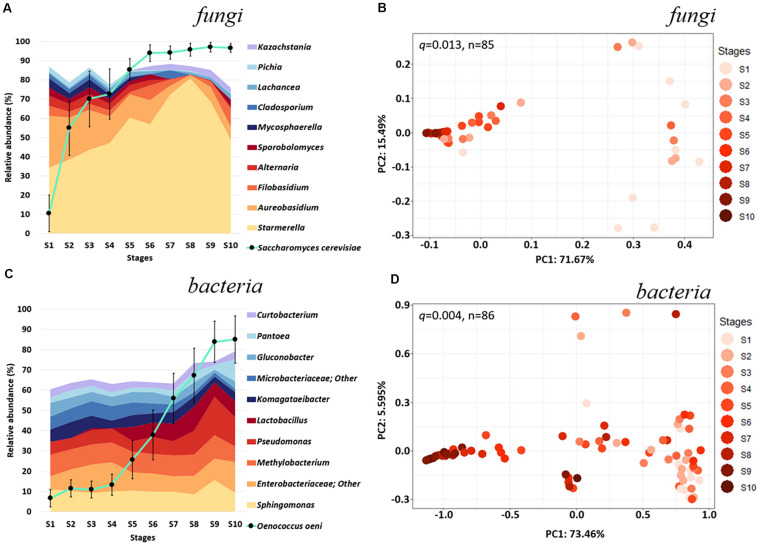
Microbial diversity across fermentation stages of Chambourcin red wine. Relative taxonomic distribution at the genus level displaying the top 10 **(A)** fungal and **(C)** bacterial community abundance throughout fermentation minus *Saccharomyces cerevisiae* and *Oenococcus oeni* (• connected by turquoise line). Taxonomic distributions for each timepoint represent the average abundance of microbial taxa detected in all samples from each fermentation stage. Weighted UniFrac PCoA for **(B)** fungal (*n* = 85) and **(D)** bacteria (*n* = 86) communities categorized by fermentation stages. Relative abundances of *S. cerevisiae* and *O. oeni* were obtained from their reads normalized to total sample reads. Error bars in **(A,C)** denote variance (Standard Error of the Mean, SEM) of *S. cerevisiae* and *O. oeni* abundances. Statistical significance for was determined by PERMANOVA (*n* = 999; Figures 1B,D and Supplementary Table 3).

In this study, our aim was to characterize the impact of hybrid grape microbial populations throughout fermentation in commercial winemaking. It is important to note that we did not ask different winemaking practices such as the addition of commercial *S. cerevisiae* and *Oenococcus oeni* and the use of sulfur dioxide during fermentation to be changed for our study in order to preserve commercial winery and vineyard practices in Pennsylvania (Supplementary Table 1). To obtain a clearer picture of the taxonomic distribution throughout the 10 fermentation stages sequence reads obtained from *S. cerevisiae* and *O. oeni* were excluded and displayed separately (Figure 1).

Analysis of fungal taxonomy highlight a few non-*Saccharomyces* yeasts were predominant throughout fermentation (Figure 1A). *Starmerella* was the most abundant non-*Saccharomyces* yeast across all stages (34.31% in S1 and 48.88% in S10), followed by *Aureobasidium* (26.97% in S1 and 6.69% in S10), *Filobasidium* (5.19% in S1 and 9.51% in S10), and *Alternaria* (5.27% in S1 and 0.98% in S10). Together, these non-*Saccharomyces* yeast account for over 71.74% and 66.06% of the non-*Saccharomyces* fungal populations in our fermentation stage 1 and 10, respectively. Interestingly, while the abundance of most non-*Saccharomyces* yeast decreased in relative abundance throughout fermentation, *Kazachstania* increased in relative abundance toward the end of fermentation from below detection level in S1 to 2.50% in stage 10 (Figure 1A). *S. cerevisiae* accounted for 18.04% of the total fungal community in stage 1 and eventually becomes the dominant species by the end of fermentation (94.42%, S10; Figure 1A).

Based on beta-diversity, fermentation of Chambourcin which included the addition of commercial *S. cerevisiae* and *O. oeni* strongly influenced the structure of the fungal community with significant dissimilarity (*q* = 0.013, pseudo-*F* = 18.58) between the initial (S1) and the end of fermentation (S10; Figure 1B). In the later stages of the fermentation, the compositions of the fungal communities from 9 wineries seem to converge, possibly due to the selective pressures of *S. cerevisiae* driving fermentation (Supplementary Table 3).

Analyses of bacterial communities indicated that among the non-*Oenococcus* taxa, *Sphingomonas* (11.02% in S1 and 9.40% in stage 10), an unidentified *Enterobacteriaceae* genus (6.61% in S1 and 15.23% in S10), and *Methylobacterium* (10.58% in S1 and 7.55% in S10) were the most abundant throughout the 10 Chambourcin fermentation stages (Figure 1C). Additionally, *Pseudomonas* (6.24 in S1 and 14.50% in S10) and *Lactobacillus* (0.12% in S1 and 7.95% in S10) showed relatively higher abundance among non-*Oenococcus* bacteria in the middle to later fermentation stages (S4–S10). Two acetic acid bacteria (AAB) genera, *Komagataeibacter* and *Gluconobacter*, were more abundant during early fermentation (5.98% and 6.85% in S1 and 7.86% and 5.42% in S2), compared to middle and late fermentation (4.30% and 3.70% in S10; Figure 1C). Relative abundances of the majority of non-*Oenococcus* bacteria seemed to fluctuate less throughout fermentation compared to the fungi (Figures 1A–C). Similar to *S. cerevisiae*, *O. oeni* accounted for only 6.67% of the total bacterial community in S1 but dominated the bacterial population by the end of fermentation (85.07%, S10; Figure 1C). This increase was not as rapid as for the commercial yeast until stage S5 where the amount of *O. oeni* grew more rapidly and took place after S7 (relative abundance > 50% of total bacteria populations), which support the survey that participating wineries added *O. oeni* during later stages of fermentation (S5–S9; Supplementary Table 1). Interestingly, beta-diversity analysis (Figure 1D) for the bacterial communities showed a high dissimilarity between the 9 different wineries, which did not converge to the same extent as for the fungal communities in the later stages of fermentation. These fermentations did, however, undergo a significant change in the composition of the bacterial community between the beginning (S1) and end (S10) of fermentation (*q* = 0.004, pseudo-*F* = 49.34; Figure 1D and Supplementary Table 3).

**FIGURE 2:**
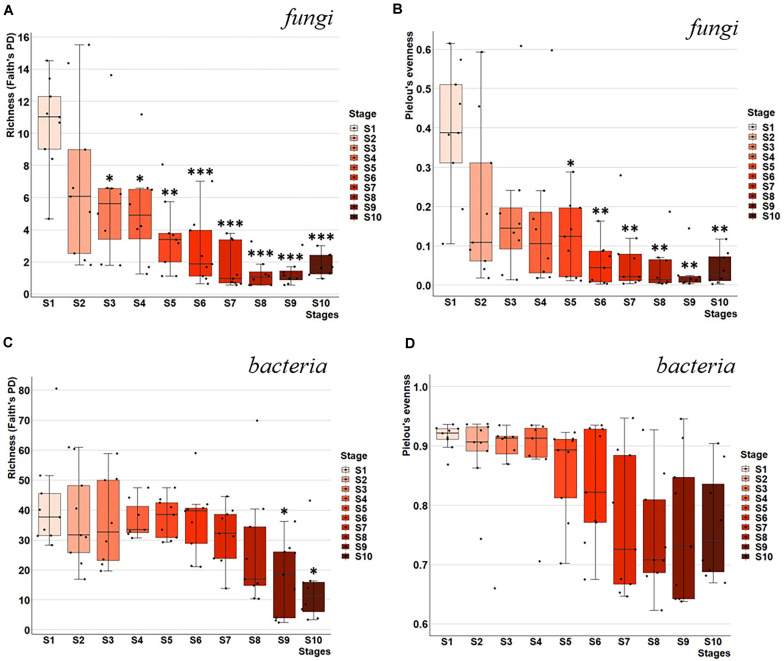
Microbial richness and evenness throughout fermentation stages of Chambourcin red wine. Rarefied alpha diversity distribution of microbial richness and evenness throughout fermentation stages for samples collected across nine wineries and ten fermentation stages. Faith’s phylogenetic diversity (PD) of richness for **(A)** fungal and **(C)** bacterial communities, and Pielou’s evenness for **(B)** fungal and **(D)** bacterial communities. Box plots represent the 1.5*IQR (Inter quartile range) divided by the square root of n, which corresponds to the 95% confidence interval. Significant differences between fermentation stages relative to stage 1 were determined using Kruskal–Wallis test with FDR-adjusted *p*-value (*q*-value). *q-value < 0.05, **q-value < 0.01, and ***q-value < 0.005.

Alpha-diversity indices, namely, Faith’s PD and Pielou’s evenness, were used to measure microbial richness and evenness within each fermentation stage, followed by a Kruskal–Wallis non-parametric test to detect significant changes between fermentation stages relative to S1 (Figure 2). We observed the highest richness (Faith’s PD = 10.59) and evenness (Pielou’s evenness = 0.40) within the fungal communities for S1, followed by a significant decline in both richness and evenness (*q* < 0.05) of the fungal communities during S3 through S5 (Figures 2A,B). This decline was characterized by a dramatic decrease in the number of different taxa followed by a succession of remaining taxa in the community (Figures 2A,B).

In contrast, bacterial communities demonstrated much higher microbial richness compared to fungal community in S1 (Faith’s PD of 42.18 vs. 10.59; Figures 2A,C). Bacterial richness and evenness did not decrease significantly in the early stages of fermentation. While both richness and evenness decreased throughout fermentation, only richness showing a significant decrease in the last two fermentation stages S9 and S10 compared to S1 (*q* < 0.05; Figures 2C,D).

### Differential Microbial Signatures Between Pennsylvania Central and East Regions

**FIGURE 3:**
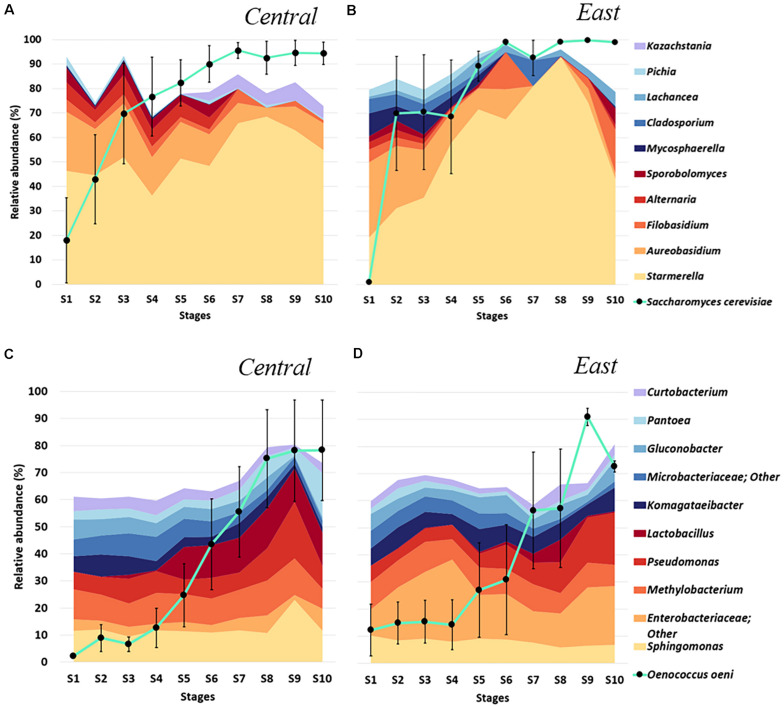
Microbial diversity across fermentation stages of Chambourcin winemaking in two Pennsylvania regions, **(A,C)** Central and **(B,D)** East. Relative taxonomic distribution at the genus level for the top 10 **(A)** fungal and **(C)** bacteria communities throughout fermentation minus *S. cerevisiae* and *O. oeni* (• connected by turquoise line). The taxonomic distribution at each timepoint represents the average abundance of microbial taxa detected in all samples from one region at each fermentation stage. Relative abundances of *S. cerevisiae* and *O. oeni* were obtained from their reads normalized to total sample reads. Error bars denote variance (Standard Error of the Mean, SEM) of *S. cerevisiae* and *O. oeni* abundances.

Besides changes in fungal and bacterial diversity obtained from ten predetermined sampling stages throughout Chambourcin fermentation (see section “Materials and Methods”), samples collected from 9 different wineries located in two different regions in PA also allowed comparison of fermentation microbiome between Central and East regions. This comparison is motivated by the research question “What is the impact of regional differences on microbial populations throughout fermentation stages in Chambourcin?”. We found that *S. cerevisiae* dramatically increased in abundance during early timepoints of fermentation (S1–S3) in samples from both regions, however, non-*Saccharomyces* yeast population showed a different pattern (Figures 3A,B). For both regions, the highest non-*Saccharomyces* yeast abundance was observed in earlier stages of fermentation; S1 and S2 for samples from the Central region and S1 for the East region. For both regions, the most abundant non-*Saccharomyces* yeast and filamentous fungi in S1 were *Starmerella* and *Aureobasidium*. However, their relative abundances and rank order differed between these two regions: *Starmerella* was the most dominant non-*Saccharomyces* yeast in the Central region (46.46% vs. 19.13% in East) while *Aureobasidium* was the most dominant species in the East region (30.69% vs. 23.99% in Central; Figures 3A,B). We further observed that samples from the East region showed higher abundances of *Mycosphaerella* and *Cladosporium* in the early stages S1 to S5 and *Lachancea* throughout the fermentation (S1–S10), while *Alternaria* was more abundant in samples from the Central region from S1 to S6. Furthermore, *Kazachstania* was only detected in samples from the Central region during middle and later fermentation stages (S5–S10; Figures 3A,B).

Among the bacteria, *O. oeni* which was the predominant species in samples from both regions beginning at the mid- fermentation stages (S7, >50% of whole bacteria populations), mirroring the impact of added commercial *S. cerevisiae* (Figures 3C,D). Similar to changes in fungal communities, the non-*Oenococcus* bacteria showed regional differences in samples collected from wineries in the Central vs. East regions of PA. Samples from the East region showed a higher abundance of *Enterobacteriaceae* related genera while *Sphingomonas* was more abundant in samples from the Central region throughout fermentation whereas *Lactobacillus* was more abundant observed in samples obtained from S5–S10 from the Central region. Interestingly, we also observed that the relative abundance of the genus *Methylobacterium* was relatively stable throughout fermentation in samples from both regions (Figures 3C,D). Taken together, these preliminary taxonomic distributions suggested distinct microbial fingerprints in Chambourcin samples throughout fermentation between the East and Central regions of Pennsylvania.

**FIGURE 4:**
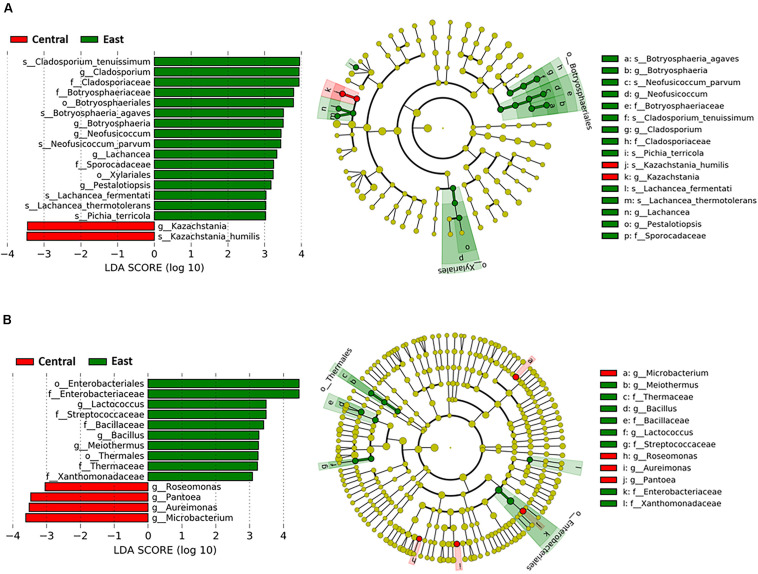
Taxa that discriminated between samples from the Central and East regions throughout Chambourcin fermentation. Linear discriminant analysis with effect size (LEfSe) analyses were performed using microbial relative abundance data. Data shown are the log_10_ linear discrimination analysis (LDA) scores following LEfSe analysis and the hierarchy of differential taxa visualized as cladograms for different taxonomic level comparisons between two regions for the **(A)** fungal, and **(B)** bacterial communities. A cut-off criterion of LDA scores >3 and *p*-value < 0.05 was used.

We employed LDA with effect size (LEfSe) to identify differences in abundance in fungal and bacterial communities at different taxonomic levels throughout fermentation stages of Chambourcin winemaking between the East and Central regions. Using an LDA score of larger than 3 and *p* < 0.05 as a cut-off, a total of 7 fungal species and 7 bacterial genera were identified as being significantly different in all samples from both regions (Figure 4). Analysis of the fungal taxa demonstrate that the abundance of *Cladosporium tenuissimum* was significantly higher in samples from the East vs. Central region across all fermentation stages, followed by *Botryosphaeria agaves*, *Neofusicoccum parvum*, *Lachancea fermentati*, *Lachancea thermotolerans*, and *Pichia terricola* (Figure 4A). In addition, within the Saccharomycetales order, the fungal species *Kazachstania humills* was identified as significantly more abundant in samples from the Central region (Figure 4A).

Based on our LDA score cut off, some notable differences in bacterial genera were evident between samples from the East and Central regions. In the East region, LEfSe identified *Lactococcus* as the most differential genus followed by *Bacillus* and *Meiothermus*. On the other hand, *Microbacterium* was the most significantly discriminating for the Central region, followed by *Aureimonas*, *Pantoea*, and *Roseomonas* (LDA > 3, *p* < 0.05; Figure 4B). Therefore, LEfSe analysis enabled identification of 14 differential fungal species and bacterial genera between the East and Central regions that were selected for further downstream analyses.

### Differences in Volatile Metabolome Throughout Fermentation Can Help Explain Differences in Chambourcin Wine Characteristics From Central and East Regions

Volatile metabolites emitted from samples collected across all fermentation stages from each of the nine wineries in the East and Central regions were analyzed by HS-SPME-GC-MS. To identify Chambourcin-associated volatile metabolites, we identified 64 core volatile compounds which were detected across all wineries in the last fermentation stage (S10) as target compounds for downstream analyses (Supplementary Tables 4–7).

**FIGURE 5:**
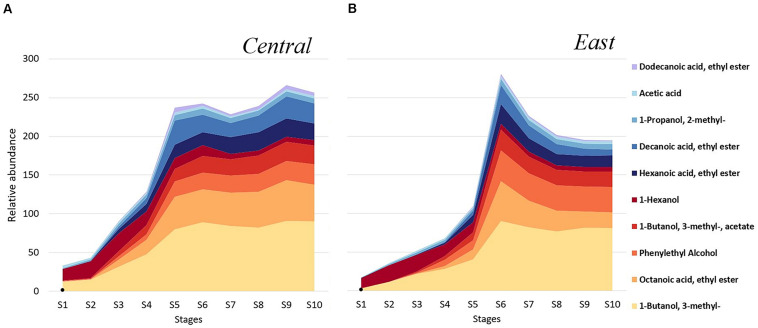
Chambourcin volatile metabolites distribution in wines from the Central (*n* = 5) and East (*n* = 4) regions. Shown are the top 10 most abundant volatile compounds in the samples throughout fermentation from the **(A)** Central, and the **(B)** East region. Relative abundance of each volatile metabolite was obtained from calculating metabolite peak area divided by IS peak areas which was acquired from each sample throughout each fermentation stage.

To explore potential regional differences in volatile profiles, we investigated the distribution of individual volatiles throughout fermentation in samples from the Central and East regions. The top 10 most abundant volatiles showed a similar pattern for both regions, with concentration increases in 1-Butanol, 3- methyl-, followed by Octanoic acid, ethyl ester and Phenylethyl Alcohol after mid-fermentation (S5–S10; Figure 5). In general, samples from the Central region were characterized by higher concentrations of Octanoic acid, ethyl ester while samples from the East region showed higher Phenylethyl Alcohol levels. Importantly, between fermentation stages S4-S6, volatile profiles began to differentiate by region. In samples from the Central region, a dramatic increase of volatile metabolites was observed moving from S4 to S5, including the ethyl esters Hexanoic acid, ethyl ester, and Decanoic acid, ethyl ester (Figure 5A). In contrast, in samples from the East region, most volatiles dramatically increased in between stage S5 to S6 and then decreased again in concentration, including alcohols, acetate and ethyl esters such as 1-Butanol, 3- methyl-, Octanoic acid, ethyl ester, 1-Butanol, 3- methyl-, acetate, Hexanoic acid, ethyl ester, and Decanoic acid, ethyl ester (Figure 5B). In contrast to most other volatiles which showed a concentration increase throughout fermentation, 1-Hexanol decreased in concentration across the fermentation stages in a similar fashion for both regions (Figures 5A,B).

**FIGURE 6:**
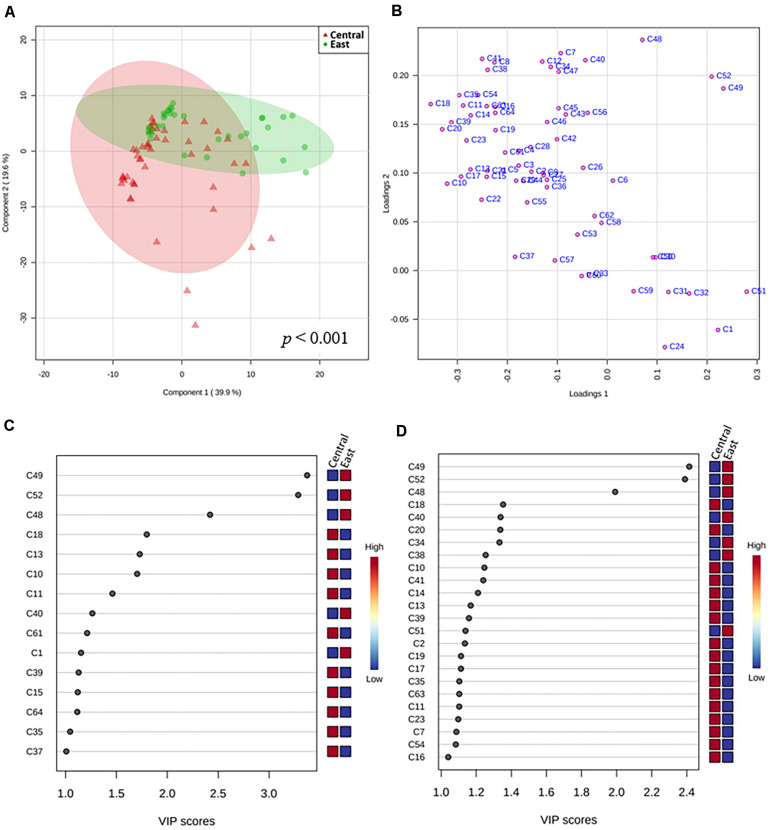
PLS-DA of volatile metabolites from fermentation samples collected from five Central and four East wineries. **(A)** Score plot showing individual samples from both regions with 95% confidence ellipses for the regions with permutation-obtained *p*-value of *p* < 0.001. **(B)** Loadings plot showing volatile metabolites; Important features in **(C)** PC1 and **(D)** PC2 based on variable importance in projection (VIP) values. The colored boxes on the right of the VIP scores indicate the relative concentrations of the corresponding metabolite in each region under study (VIP > 1). Note: Volatile metabolite codes are provided in Supplementary Table 5.

It is important to note that volatiles are produced continuously during Chambourcin wine fermentation. Regional microbial communities present on grape berries and in wineries have been shown to be an important contributor to sensory wine characteristics. To better identify volatile metabolites generated during fermentation that could be associated with specific wine-producing regions, a targeted PLS-DA model was created using the 64 core Chambourcin volatile metabolites measured from 83 fermentation samples obtained from the Central and East regions (Figure 6). A validated PLS-DA was obtained for our model based on permutation tests (*p* < 0.001), however, the goodness of fit and goodness of prediction indicated moderate predictive accuracy (2 components, *R*^2^ = 0.39, *Q*^2^ = 0.18). Using a score plot to visualize the separation of samples from driven by two regions, the first two model components captured 39.9% and 19.6% of the total variance, respectively, (Figure 6A and Supplementary Figures 2, 3). Our interpretation of the data suggested separation of features in the two regions to some degree: fermentation samples collected from the East region showed less variation compared to the Central region, as indicated by the size of confidence ellipses. The loadings plot in Figure 6B shows that most esters were located in the top left quadrant demonstrating high positive correlation to samples obtained from later stages of fermentation (S7–S10) from both regions. Separation along the first axis was mainly driven by differences in Hexanal (C51), (E)-2-Hexenoic acid (C49), Butanoic acid, methyl ester (C1), and Heptanal (C52) concentrations on the positive PC1 axis and Decanoic acid, ethyl ester (C18), Butanedioic acid, diethyl ester (C20), and Ethyl (S)-(-)-lactate (C10) on the negative PC 1 dimension. Furthermore, the negative PC2 axis was mainly driven by higher concentrations of 3-Buten-2-ol, 2-methyl- (C24), C1, 2-Hexen-1-ol, (Z)- (C32), and 3-Hexen-1-ol, (Z)- (C31; Supplementary Table 5). Finally, using VIP values of over 1 on either of the first two PCs as a cut-off level for metabolite discrimination between regions, the most differential volatiles (VIP > 2) were (E)-2-Hexenoic acid (C49), Heptanal (C52), and Heptanoic acid (C48), which all showed higher concentrations in samples from the East region (Figure 6C). Additional metabolites with VIP scores of more than 1.5 included Decanoic acid, ethyl ester (C18), Octanoic acid, methyl ester (C13), and Ethyl (S)-(-)-lactate (C10), which were all found in higher levels in samples from the Central region (Figures 6C,D). Using a regional PLS-DA model and VIP scores of larger than 1 as cut-off, 29 volatile metabolites from the 83 fermentation samples were selected for downstream correlation analysis.

### Fungal Taxa Strongly Associate With Volatile Metabolites in the East Region While Volatile Metabolome in the Central Region Associates With Bacterial Taxa

**FIGURE 7:**
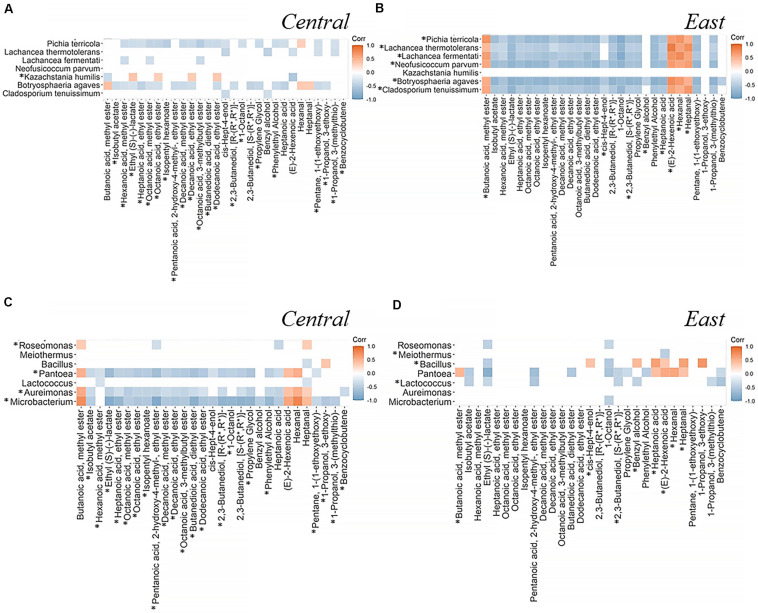
Spearman’s correlation analysis between significantly different microorganisms identified by LEfSe and volatile metabolites identified by PLS-DA. Data shown here are the correlation coefficients between seven fungi **(A,B)** or seven bacteria **(C,D)** and 29 volatile metabolites across fermentation stages in the Central **(A,C)** and East **(B,D)** regions. Positive correlations on the heatmap are represented by orange and negative correlation are in gray. Spearman’s correlation significance indicated by coefficient with *q*-value < 0.05 (coefficients with *q* > 0.05 are shown in white block). The comprehensive correlation heatmaps of microbial communities and volatile metabolites are provided in the Supplementary Figure 4. *highlighted the features with higher abundance for the indicated region.

In this section, we aim to identify associations between microbial communities and volatile metabolites that differentiate between regions by performing a quantitative Spearman’s correlation analysis. Using all significant correlation coefficients (*q* < 0.05) between seven fungal species and seven bacterial genera and 29 volatile metabolites, we created a correlation heatmap to help us explain microbiome-metabolome differences of associations that are characteristic of Central and East regions (Figure 7 and Supplementary Figure 4). Most significant associations between microbial communities and volatile metabolites were negatively correlated (Fungi, 85% in the Central and 88% in the East; Bacteria, 83% in the Central and 67% in the East; Figure 7). Among those associations, we could observe that East region was characterized by strong associations between metabolites and the fungal population, while the Central was more characterized by bacterial associations. Nevertheless, it should be noted that microorganisms did not necessarily produce the correlated compound themselves, but rather may influence the overall production or consumption by the community.

As for the fungal community, *Neofusicoccum parvum* did not demonstrate a significant correlation in the Central region but showed a strong microbiome-metabolome correlation in the East region (*q*-value < 0.05). On the other hand, *Lachancea thermotolerans* was negatively correlated with *cis*-Hept-4-enol and Benzyl alcohol in the Central region but there were no significant correlations of these two metabolites in the East region. Notably, (E)-2-Hexenoic acid showed a positive correlation with *L. thermotolerans* in the East, which was opposite from the Central. Interestingly, *Kazachstania humilis* was only detected in the Central region and demonstrated opposing patterns of correlations compared with other six differential fungi. For instance, it demonstrated positive correlations with Ethyl (S)-(-)-lactate, Octanoic acid, ethyl ester and Decanoic acid, ethyl ester while negative correlations were shown in other six fungal species (Figures 7A,B).

Analysis of microbiome-metabolome interactions focused on bacterial communities highlight a total of seven different bacterial genera with distinct patterns of associations with 29 volatile metabolites from the East and Central region. For instance, genera *Pantoea*, *Aureimonas*, and *Microbacterium* were key features in the Central region where as *Bacillus* and *Lactococcus* were key features in the East (Figures 7C,D). Meanwhile, there were no significant correlations shown between *Meiothermus* and metabolites in the Central region, instead, this genus showed negative association with (E)-2-Hexenoic acid in the East region. Interestingly, *Pantoea* was negatively correlated with Heptanoic acid in the Central region but positively correlated with this compound in the East. In the Central region, *Bacillus* negatively correlated with Heptanal but the opposite correlation was found in the East. Unlike the fungal community where most species exhibited similar correlation patterns, it seemed to be more common that bacterial taxa exhibit negative associations with metabolites within a region, for example, Heptanal and 1-Propanol, 3-ethoxy- in the Central region and Benzyl alcohol and (E)-2-Hexenoic acid in the East region.

## Discussion

### Changes in Microbial Communities Throughout Chambourcin Fermentation Provide Insights Into the Microbiome of Interspecific Grape Varieties

Recent studies have demonstrated that microbial populations present on grape berries, in wineries, and throughout fermentation can impact final wine characteristics and contribute to *terroir* (Bozoudi and Tsaltas, 2016). While most studies on wine microbiome focus on *V. vinifera* grape varieties, the focus of this study is on Chambourcin, an interspecific hybrid grape variety widely grown in Pennsylvania and the Midwest and Eastern regions of the United States. Hybrid grapes such as Chambourcin are of great interest to the wine industry due to their resistance to diseases and adaptability to changing climates (González-Centeno et al., 2019; Santos et al., 2020). The ability to understand how microbial populations on hybrid grapes impact specific wine characteristics could help grape growers and winemakers monitor spoilage microorganisms on berries, prevent potential wine faults, and control final wine quality. In our study, alpha and beta diversity analyses of Chambourcin microbiome demonstrate decrease diversity of native microbial populations throughout fermentation. It is important to note that all participating wineries added commercial *S. cerevisiae* and *O. oeni* as oppose to spontaneous fermentation which is one factor that can impact microbial diversity.

The use of commercial *S. cerevisiae* and *O. oeni* during winemaking is a common practice in winemaking. Commercial *S. cerevisiae* is added relatively early during fermentation to ensure a complete fermentation and reduce the risk of stuck or sluggish fermentation. However, this practice has been shown to significantly decrease microbial diversity during fermentation (Bokulich et al., 2016; Dimitrios et al., 2019) resulting in lower complexity of wine important aroma compounds (Steensels and Verstrepen, 2014; Bozoudi and Tsaltas, 2016). Previous literature on wine microbiology emphasizes the role of fungal community, especially yeasts in winemaking. However, diversity analysis in our study demonstrate that non-*Oenococcus* bacterial populations maintain a certain level of population evenness without a significant dominant taxon throughout fermentation. Our results deviate from prior literature that reported the increased abundance of *Gluconobacter, Komagataeibacter*, or *Enterobacteriaceae* during fermentation. The difference in distribution patterns of bacterial communities and other wine-related studies gives a distinct bacterial signature of Pennsylvania Chambourcin fermentation (Bokulich et al., 2016; Marzano et al., 2016; Jiang et al., 2020).

Native fungal populations present on grape berries are important for winemaking and for final wine quality (Jolly et al., 2014). Despite the common practice of adding commercial *S. cerevisiae* to fermenting must, native fungal populations present during early stages of fermentation have been reported to establish a microbial fingerprint that influences production of important wine volatile compounds (Steensels and Verstrepen, 2014; Andorrà et al., 2019). For example, fungal population in Cabernet Sauvignon must from four different California regions were dominated by filamentous fungi, such as *Cladosporium* spp., *Botryotinia cinerea*, and *Penicillium* spp. (Bokulich et al., 2014). For Cannonau grape must samples collected from four localities in Sardinia, Italy the fungal community was dominated primarily by the genera, *Aureobasidium*, *Alternaria*, and *Hanseniaspora* (Mezzasalma et al., 2017). In our study on interspecific Chambourcin grape fermentation, *Starmerella*, *Aureobasidium*, *Filobasidium*, and *Alternaria* dominated fungal population during early fermentation (first 4 days), after which *S. cerevisiae* became the dominant species.

The abundance of native fungal populations on PA Chambourcin could be a characteristic differentiator of final wines. One specific example is observation of high levels of *Starmerella bacillaris* (synonym *Candida zemplinina*, abbreviated *Starm. bacillaris*; Sipiczki, 2003) throughout Chambourcin fermentation (except *S. cerevisiae*) in wine grapes must and this could be an early indicator of wine quality. *S. bacillaris* is frequently found on overripe grape berries because of its fructophilic character indicative of high sugar content in grapes during harvest (Englezos et al., 2017; Horváth et al., 2020). In addition, an interesting application for *Starm. bacillaris* is as a biocontrol fungus for preventing *Botrytis cinerea* infection which is often associated with overripe grapes and is a challenge grapevine in temperate climates (Lemos et al., 2016). The presence of high levels of *Starm. bacillaris* in our fermentation microbiome analysis could explain why we did not detect *B. cinerea* but only small amount of *Botrytis caroliniana* in the grape must (data not shown). On the other hand, adding high level of *Starm*. *bacillaris* followed by *S. cerevisiae* in the beginning of fermentation through sequential inoculation of wine grapes with increased maturity (high sugar content) has been highlighted as a strategy to reduce ethanol levels (Englezos et al., 2017; Goold et al., 2017). Furthermore, this inoculation practice can increase desirable compounds such as glycerol. In addition, co-inoculation of *Starm. bacillaris* and *S. cerevisiae* resulted in production of target volatile metabolite such as 2-phenylethanol, 1-hexanol, 2-methyl 1-propanol, and acetic acid that contributed to the sensory properties of Montepulciano red wines, which could be one of factors explaining our volatile compound compositions during early fermentation (Tofalo et al., 2016). In support of this, high levels of *Starm. bacillaris* observed in our study during early stages of fermentation likely contributes to microbial *terroir* and wine quality.

Next, *Aureobasidium* (*A. pullulans*) was the second most abundant fungus during early fermentation in our study. *A. pullulans* is well-known for the production of amylase and β-glucosidase enzymes that aid in the release of glycosylated aroma volatiles which has been shown to improve aroma perception of red wines (Baffi et al., 2013; Englezos et al., 2018). Our volatile analyses of Chambourcin samples throughout fermentation detected higher levels of phenylethyl alcohol, 3-methyl-1-butanol, and octanoic acid, ethyl ester which could be explained by previous studies that characterize the role of *A. pullulans* on production of typical flavor compounds of red wine (Verginer et al., 2010; Bozoudi and Tsaltas, 2018). From a spoilage standpoint, the antimicrobial activity of *A. pullulans* against spoilage fungi such as *B. cinerea*, or the bacterial pathogen *Staphylococcus aureus* could offer some protection during winemaking (Cho et al., 2015; Bozoudi and Tsaltas, 2018). On the other hand, considering the regionality of colonization, *A. pullulans* has been found in high abundance at harvest in Italy, Spain, Australia, South Africa and Canada (Wang et al., 2015; Bozoudi and Tsaltas, 2016) while it was not detected at harvest of Merlot, Cabernet Sauvignon and Cabernet Franc grapes in the Bordeaux area, France (Renouf et al., 2005). Therefore, it might indicate that the Central and East PA regions are also one of the areas suitable for *A. pullulans* taking its role during wine fermentation.

An interesting observation in our study is the persistence of *Filobasidium* (*F. magnum*) throughout fermentation (S1–10). *Filobasidium magnum* was found to be present on *V. vinifera* grape berries but not in the unfiltered wine (Kačániová et al., 2020) indicating that this species could play a role before alcoholic fermentation begins. In addition, *F. magnum* has also been isolated from apples and pears (Glushakova and Kachalkin, 2017). While previous studies have demonstrated that *F. magnum* is a ubiquitous in vineyards and on grape berries, the impact of *F. magnum* on production of wine volatile metabolites is not known. We hypothesize that its presence on different fruits and in high sugar environments would make this genera good candidates to study for potential use as starters in winemaking (Lee et al., 2011).

During wine fermentation, LAB and AAB are two main bacterial groups that are known to impact final wine characteristics (Bozoudi and Tsaltas, 2016). In this study, *Sphingomonas*, *Enterobacteriaceae*, *Methylobacterium*, *Pseudomonas, Lactobacillus*, and *Komagataeibacter* were the most abundant bacterial genera detected during Chambourcin fermentation. Previous studies demonstrate that *Sphingomonas* and *Methylobacterium* constitute 6–13% of the total bacterial population present on different *V. vinifera* grapes (Bokulich et al., 2013; Kántor et al., 2017) while *Sphingomonas* was shown to positively correlate with fermentation rate (Brix hr^1^; Bokulich et al., 2016). Likewise, *Enterobacteriaceae* was found to be abundant in different *V. vinifera* grape musts undergoing either spontaneous or inoculated fermentations showing its universal patterns in the red wine system (Pinto et al., 2015; Bokulich et al., 2016).

Our study showed that *Lactobacillus* was more abundant in later stages of fermentation (after S5) which could be due to higher tolerance to alcohol concentrations (Gold et al., 1992). This genus is one of the most relevant LAB in winemaking known for the production of volatile compounds that influence wine sensory attributes such as 2,3-Butanedione (Diacetyl) with a buttery, creamy aroma, geranium-smelling 2-Ethoxy-3,5-hexadiene, and vinegar-smelling acetic acid (Inês and Falco, 2018). On the contrary, AAB are commonly considered to be wine spoilage bacteria due to the production of acetaldehyde and acetic acid (Mamlouk and Gullo, 2013). In our study, two genera, *Komagataeibacter* and *Gluconobacter*, within the family of AAB were abundant from the first to the middle stage (S5), though they existed throughout fermentation. *Komagataeibacter* has been shown to decrease wine quality due to its ability to oxidize sugars and sugar alcohols (D-glucose, glycerol, and ethanol), excrete exopolysaccharides, and tolerate high acetic acid concentrations, leading to a high persistence of this bacteria in fermentation environments (Bokulich et al., 2013; Zhang et al., 2017). Likewise, Gluconobacter was reported to influence wine quality by oxidizing glucose and ethanol to acids (Drysdale and Fleet, 1988). According to our survey, four wineries (three in the Central and one in the East region) added sulfur dioxide during early fermentation stages (S1–S3; Supplementary Table 1). However, the abundances of these two AAB genera seem to be less affected by sulfur dioxide. This observation could be due to the concentration of active sulfites or the existence of higher sulfur dioxide-tolerating strains (Drysdale and Fleet, 1988; Andorrà et al., 2008).

In summary, high-throughput sequencing and biodiversity analysis demonstrate several dominant genera throughout fermentation of Chambourcin grapes that could contribute to the unique characteristics of Pennsylvania Chambourcin red wines. This highlights potential factors that can affect condition of grapes at harvest such as overripening or presence of Botrytis infection. It would be of interest in the future to compare microbial communities on *V. vinifera* grapes to other varieties within the same vineyard within the same harvest.

### Wine Fermentation Microbiome and Dominant Taxa Identified by LEfSe Suggest Regional Differences Between Chambourcin From the Central and East Regions

Regional characteristics of wines could be influenced by microbial *terroir*, i.e., the overall fungal and bacterial distribution patterns present on grapes and throughout fermentation (Gilbert et al., 2014; Capozzi et al., 2015; Bokulich et al., 2016; Liu et al., 2019). Here, LEfSe analysis identified specific bacterial and fungal species that differed between samples collected from two different wine-growing regions in Pennsylvania. *Cladosporium* (*C. tenuissimum*) was found to be the most abundant fungi in samples from the East, followed by *Botryosphaeria* (*B*. *agaves*), and *Neofusicoccum* (*N. parvum*). The higher abundance of these three genera on PA Chambourcin could be explained by differences in climate, as prior studies reported that wet weathers or free water can lead to germination of fungal conidia (Niekerk et al., 2006; Espinoza et al., 2009; Wang et al., 2015). Although the presence of genetic material (DNA) does not indicate the presence of grape vine infection (Taylor et al., 2014), these filamentous fungi are considered plant pathogens which could result in poor grape quality and spoilage influencing final wine quality (Latorre et al., 2011; Pitt et al., 2012; Lorenzini et al., 2015).

Among the yeast community, *Lachancea* (*L. fermentati*), *L. thermotolerans*, and *Pichia* (*P. terricola*) also showed regional differences and were more abundant in samples from the East. First, *L. fermentati* and *L. thermotolerans* are two species most frequently isolated from grape must and wine fermentation processes (Porter et al., 2019b). Studies reported that *L. fermentati* showed a high SO_2_ tolerance (20 mg/L total SO_2_) and high fermentation activity in monoculture. In addition, the presence of *L. fermentati* is frequently associated with higher levels of Isobutanol and Isobutyric acid in Muscat wines and mixed-fermentations of *L. fermentati* with *L. thermotolerans* enhances production of monotepenes such as linalool and geraniol leading to perceivable aroma contribution in wine (Porter et al., 2019a,b). Furthermore, *L. thermotolerans* when co-cultured with *S. cerevisiae* was shown to contribute to the reduction in acetic acid and increase in Phenylethyl Alcohol and glycerol levels, which may help explain the higher Phenylethyl Alcohol content in samples from the East region (Figure 5; Ciani et al., 2006; Kapsopoulou et al., 2007; Comitini et al., 2011; Gobbi et al., 2013). Other than the direct production of volatile metabolites, *P. terricola* was reported to produce the extracellular enzyme, beta-glucosidase altering the sensory perception of Muscat wine wines (González-Pombo et al., 2011). Although yeasts of the genus *Pichia* has been previously shown to not persist past early stages of fermentation (Fleet et al., 1984), our study showed the persistence of *Pichia* throughout middle stages of fermentation (S1–5) suggesting a possible role in the ecology of wine fermentation in the East region. In samples from the Central region, *Kazachstania* (*K. humilis*) was the most discriminative non-*Saccharomyces* yeast. Previous studies mentioned that *K. humilis* is able to produce ethyl acetate, acetaldehyde, and ethanol during kaoliang and sourdough fermentation (DiCagno et al., 2014; Lai et al., 2019). Although not shown in grape fermentations, *K. humilis* appears to be able to influence the bacterial population and produce several volatile metabolites during different food fermentation, giving the potential role on wine characteristics.

Among the bacterial community, *Lactococcus* was the most differentially abundant genus in the East region followed by *Bacillus*. A member of the LAB *Lactococcus* has been reported to produce high-level diacetyl responsible for buttery flavor during dairy fermentation and has high association with carbonyl compounds in rice wine (Hugenholtz et al., 2000; Liu et al., 2015). Furthermore, it has been reported to carry out MLF and produce lactic acid and esters during white wine fermentation (Kurane and Ghosh, 2012). As for *Bacillus*, this genus has been found to have a positive correlation with pyrazines which are associated with herbal and vegetal aromas (Ren et al., 2019). In the Central region, *Microbacterium* was the most differentially abundant genus followed by *Aureimonas*, *Pantoea*, and *Roseomonas*. Although these grape epiphytic bacteria *Microbacterium, Aureimonas*, and *Pantoae* has been found on grapevine, leaves and grape must, their contribution to aroma attributes in winemaking is not known (Madhaiyan et al., 2013; Godálová et al., 2016; Salvetti et al., 2016; Liu et al., 2020). Additionally, *Roseomonas* species are reported to produce bacteriochlorophyll-a, a bacterial photosynthetic pigment, which might have negative impact on wine color (Hyeon and Jeon, 2017). Collectively, these wine-associated bacteria are strong candidates playing a key role in shaping microbial *terroir* of two Chambourcin growing regions of PA. As the use of Chambourcin and other hybrid varieties develops, monitoring microbial signatures may be important to maintaining regional qualities of final wines.

### Differences of Volatile Metabolome Suggest Regionality of Chambourcin Wines and Key Metabolite Provided by PLS-DA Highlight Regional Characteristics of Chambourcin Fermentation Processes

Volatile aroma compounds contribute to the sensory properties and perception of wine (Vilanova et al., 2010). In our study, fermentation-derived volatile metabolites increased in concentration after stage 2 (=48 h) and reached a plateau around stage 6 (=7 days). Our results on accumulation of volatile alcohols and esters agree with previous literature reporting esters and higher alcohols as common wine fermentation metabolites. In Cabernet Sauvignon, volatile alcohols and esters increased in concentration in the first 24 to 36 h of fermentation reaching an exponential phase (72–84 h), after which they either decrease slightly or remain constant (Callejón et al., 2012). In addition, 1-Hexanol levels were previously reported to increase after 24 h and then decreased after 48 h, similar to our findings (Callejón et al., 2012); 1-Hexanol has been previously reported to correlate with green vegetable aroma (Zhang et al., 2015).

Following individual volatile metabolites throughout fermentation provides a baseline for aroma compounds in Chambourcin red wine which to date has not been documented. For example, 1-Butanol, 3-methyl- (Isoamyl alcohol) was the most abundant compound in samples from both regions after S6 (mid fermentation; 7 days after crush). Wine samples (S10) from the Central region were more abundant in Octanoic acid, ethyl ester (Ethyl Octanoate) whereas samples from the East region showed higher concentration of Phenylethyl Alcohol. Wine volatile profiles with abundant esters and higher alcohols are common to red wine fermentations (Morakul et al., 2013), and moreover, Isoamyl alcohol and Phenylethyl alcohol are reported as inherent alcohols for red wines produced from Cabernet Franc, Cabernet Sauvignon, Meritage, Merlot, Pinot noir, and Syrah, indicating similarity between the wines made from interspecific Chambourcin and *V. vinifera* grapes (Bejaei et al., 2019). At higher concentrations at 430 mg/L and more, isoamyl alcohol could impact wine quality negatively while Phenylethyl alcohol may contribute to the floral character in wines, however, a clear contribution of individual compounds to wine aroma is difficult due to the complexity of the volatile wine matrix (Morakul et al., 2013; De-La-Fuente-Blanco et al., 2016). Furthermore, we identified a number of acetate and ethyl esters present at high concentrations such as 1-Butanol, 3- methyl-, acetate, Decanoic acid, ethyl ester, and Hexanoic acid, ethyl ester, all commonly reported in *V. vinifera* red wines (Bejaei et al., 2019). Interestingly, Octanoic acid, ethyl ester and Decanoic acid, ethyl ester as the top abundant esters were reported as aroma enhancer compounds in Cabernet Sauvignon and Cabernet Gernischt wines (Welke et al., 2014); a similar positive contribution to wine aroma could be suggested for the Chambourcin red wines in our study. Acetic acid was the most abundant acid in Chambourcin red wine, however, well below any potential legal limits of 1.4 g/L (The standards of identity, 2016) and similar to red wines in general.

To further understand the difference of volatile profiles between regions, we use a PLS-DA modeling to discriminate differential volatile metabolites throughout fermentation. Although the power of predictive classification was low, our results suggest that the volatile profiles of wines from the two regions were distinct. Acids and aldehydes were found to be key features for regional differentiation. Higher abundance of volatile acids and aldehyde in wines seem to be caused by the activity of spoilage bacteria (Bartowsky, 2009). Future work will focus on these metabolites as potential features of wine quality Interestingly, comparing with a Spanish study of *V. vinifera* red wines that found the most differential volatile metabolites between regions to be higher alcohols (López-Rituerto et al., 2012), acids and aldehydes which were discriminated between Pennsylvania regions in our study could be a sign of *terroir*.

### Regionality Demonstrated Different Patterns of Associations Between Microbial Taxa and Volatile Metabolites Throughout Fermentation Processes

Do changes in microbial communities and volatile metabolites reflect *terroir*? Volatile metabolites’ secretion from the grape matrix or conversion from other precursors by different enzymatic activities of microorganisms lead to fluctuations in their relative abundances (López et al., 2015; Dimitrios et al., 2019; Noecker et al., 2019). Therefore, using Spearman’s correlation and focusing on the relative abundances of key differential features in microorganisms and volatile metabolites characteristic of each region, we can begin to understand how microbial *terroir* influences regional Chambourcin wine volatile profiles (Li et al., 2018). Although, the interpretation of correlation is challenging in a complex fermentation system such as winemaking, we propose the associations of microbiome and metabolome as a “fingerprint” within Pennsylvania Chambourcin red wine (Steuer et al., 2003). Thus, microbiome-metabolome correlation heatmaps (Figure 7 and Supplementary Figure 4) could reveal previously unknown associations to help guide downstream analysis.

Our results support previous studies that highlight the importance of *microbial terroir* on regional identities of red wines (Bokulich et al., 2014; Knight et al., 2015). Specifically, microorganisms with a higher degree of correlation or a different correlated pattern between regions can be suggested to have a more important role on the regional aroma profile of Chambourcin fermentations in the certain region. Analysis of fungal community suggest that *K. humilis* is strongly correlations with specific ester metabolites in the Central than the East showing its influence during fermentation in the Central region. Furthermore, a study has also mentioned its ability to produce Octanoic acid, ethyl ester (Ethyl octanoate) and Decanoic acid, ethyl ester (Ethyl decanoate; Xu et al., 2019).

On the other hand, regional differences could be attributed to microbial interactions. It has been mentioned that regionality of soil microbiota and grapevine’s epiphytes and endophytes could modulate the abundances of other microorganisms as well as grape itself and eventually influence the quality of final wine products (Gilbert et al., 2014). In other words, the compositions of regional fungal and bacterial communities with their differential relative abundances could affect wine phenotypes through synergistic interactions (Roullier-Gall et al., 2020). For instance, *L. thermotolerans* has been reported to emulate MLF or pH reduction by co-inoculation with different yeasts or LAB (Morata et al., 2018). However, the interactions between other microorganisms and the ability to produce aroma compounds remain to be evaluated. Moreover, the biosynthesis of volatile metabolites have been studied to be microbial strains or species level-related, including acetaldehyde, acetoin, and acetic acid (Swiegers et al., 2005; Capece et al., 2013). Therefore, the differences of correlation patterns can be due to the underlying regional fungal strains and bacterial species which were limited identified based on our sequencing analyses. In both regions, our observation shows that certain fungi and bacteria correlated with specific volatile compounds displaying similar correlation patterns (Figure 7B). We hypothesize that this could be caused by exogenous factors such as environmental stress on hybrid grape integrity, procedural differences in winemaking, and hybrid grape ecology (vs. *V. vinifera*), especially for red wines which has been shown to be more easily influenced by these factors compared to white wines (Bubeck et al., 2020). Accordingly, it is possible that these exogenous factors other than fermentation drives changes in microbial composition and volatile metabolite production.

To our best knowledge, this is the first study to characterize the microbiome and volatile metabolome throughout fermentation in commercial wineries located in Pennsylvania using Chambourcin hybrid grapes as a research model. Characterization of microbial communities and the volatile metabolome could provide winemakers with data-based knowledge and expand our understanding of hybrid grape varieties and their final wine characteristics, wine spoilage control, and sustainable viticulture (Bozoudi and Tsaltas, 2016; Coia and Ward, 2017; Santos et al., 2020). Additionally, regional wine typicality or wine *terroir* together with higher wine quality typically results in increased consumer acceptance and appreciation (Belda et al., 2017). Therefore, the extent to which individual microorganisms and microbial *terroir* and the stability of communities preserve the regionality of wine aroma profiles over time still needs to be evaluated. Thus, through targeted microbial manipulation coupled with culture-dependent approaches as well as human and instrumental sensory analyses, it is possible to provide a comprehensive and robust approach to improve wine’s quality (Kontkanen et al., 2005; Swiegers et al., 2005; Cadot et al., 2012).

## Data Availability Statement

All raw sequence data related to this study are available in the Sequence Read Archive (SRA) 877 under (The National Center for Biotechnology Information, NCBI) BioProject (Accession No. 878 PRJNA655761).

## Author Contributions

HW and JW designed the experiment. HW prepared and analyzed the samples. HW produced the figures, tables, and wrote the manuscript. JW and DC advised on the microbiome analyses. HH advised on the volatile metabolite analyses. JW, HH, and DC advised on the figure and table production as well as the manuscript preparation. All authors contributed to the article and approved the submitted version.

## Conflict of Interest

The authors declare that the research was conducted in the absence of any commercial or financial relationships that could be construed as a potential conflict of interest.

## Footnotes

1 www.rstudio.com
