# Supplementary material for: Characterization of Microbial Dynamics and Volatile Metabolome Changes During Fermentation of Chambourcin Hybrid Grapes From Two Pennsylvania Regions
Source: Front Microbiol. 2021 Jan 11;11:614278. doi: 10.3389/fmicb.2020.614278 (PMC7829364; doi:10.3389/fmicb.2020.614278)
Supplement: Supplementary file 1 [file Table_1.PDF]

Supplementary Table 1. The survey results from each participated winery. The results demonstrated the timepoint that the winery add commercial yeast and bacterial during fermentation processes. NA, no given answers or no action applied.

Winery	Timepoints when winemaking practices are applied		
	S. cerevisiae	*O. oeni*	sulfur dioxide
PA19_01	S1	S6	NA
PA19_02	S1	S8 – S9	NA
PA19_03	S4	S8	S1
PA19_04	S3	S4	S3
PA19_05	S2	S5	S2
PA19_06	S1	S5	NA
PA19_07	S3	S7	S1
PA19_08	S1	S7	NA
PA19_09	S4	S9	NA
