# Supplementary material for: Characterization of Microbial Dynamics and Volatile Metabolome Changes During Fermentation of Chambourcin Hybrid Grapes From Two Pennsylvania Regions
Source: Front Microbiol. 2021 Jan 11;11:614278. doi: 10.3389/fmicb.2020.614278 (PMC7829364; doi:10.3389/fmicb.2020.614278)
Supplement: Supplementary file 2 [file Table_2.PDF]

Supplementary Table 2. Summary of fermentation stages and the collection timeline of samples.

Stage	Timeline (day)	Process
S1	0	Crush (de-stemming)
S2	1	Must 1
S3	2	Must 2
S4	3	Must 3
S5	4	Early fermentation (Early)
S6	7	Middle fermentation (Mid)
S7	1	Late fermentation (Late)
S8	13	End fermentation (End)
S9	16	Malolactic fermentation (MLF)
S10	20	Wine (before racking and filtration)
