# Supplementary material for: Characterization of Microbial Dynamics and Volatile Metabolome Changes During Fermentation of Chambourcin Hybrid Grapes From Two Pennsylvania Regions
Source: Front Microbiol. 2021 Jan 11;11:614278. doi: 10.3389/fmicb.2020.614278 (PMC7829364; doi:10.3389/fmicb.2020.614278)
Supplement: Supplementary file 3 [file Table_3.PDF]

Supplementary Table 3. PERMANOVA pairwise comparisons of weighted UniFrac distance between different fermentation stages.

Fermentation stages		Fungal community			Bacterial community		
		Weighted UniFrac distance					
Group 1	Group 2	Sample size	pseudo-F	*q*-value	Sample size	pseudo-F	*q*-value
S1	S2	18	3.828038	0.063	18	0.484588	0.91
	S3	17	5.85518	0.023	17	0.704059	0.773
	S4	17	8.831802	0.02	16	0.987734	0.444
	S5	18	17.06378	0.008	18	2.914561	0.073
	S6	18	20.58302	0.008	18	5.311431	0.036
	S7	18	19.30636	0.008	18	15.91872	0.004
	S8	17	18.67642	0.008	18	15.77635	0.004
	S9	17	18.95411	0.008	18	57.07419	0.006
	S10	17	18.58474	0.013	17	49.34438	0.004
S2	S3	17	0.216045	0.848	17	0.167136	1
	S4	17	0.790607	0.608	16	0.359458	0.949
	S5	18	2.693065	0.151	18	1.632211	0.24
	S6	18	4.444437	0.051	18	3.505624	0.057
	S7	18	3.770056	0.063	18	11.14037	0.006
	S8	17	4.154183	0.052	18	11.68625	0.004
	S9	17	4.298169	0.052	18	38.29292	0.004
	S10	17	4.100239	0.062	17	33.48876	0.004
S3	S4	16	0.226145	0.848	15	0.260439	0.977
	S5	17	1.555439	0.345	17	1.456733	0.24
	S6	17	3.149714	0.038	17	2.994546	0.08
	S7	17	2.513396	0.151	17	9.651533	0.006
	S8	16	2.996994	0.033	17	10.05062	0.004
	S9	16	3.140037	0.008	17	33.56183	0.004
	S10	16	2.948056	0.038	16	29.28798	0.004
S4	S5	17	0.708215	0.739	16	1.167715	0.366
	S6	17	2.314253	0.061	16	2.53004	0.121
	S7	17	1.674486	0.323	16	8.590384	0.01
	S8	16	2.291145	0.048	16	8.943407	0.004
	S9	16	2.439493	0.02	16	32.6824	0.004
	S10	16	2.245295	0.052	15	28.38588	0.008
S5	S6	18	3.864431	0.116	18	0.50562	0.773
	S7	18	1.2274	0.388	18	3.815476	0.073
	S8	17	4.755415	0.069	18	4.919746	0.041
	S9	17	5.523377	0.052	18	17.54416	0.004

	S10	17	4.845205	0.074	17	15.52772	0.01
S6	S7	18	0.594074	0.848	18	1.646778	0.266
	S8	17	0.266048	0.739	18	2.491743	0.158
	S9	17	0.79299	0.633	18	10.06613	0.012
	S10	17	0.378298	0.812	17	9.019105	0.016
S7	S8	17	0.749766	0.812	18	0.403942	0.773
	S9	17	1.058671	0.606	18	4.120162	0.073
	S10	17	0.701296	0.812	17	3.819443	0.073
S8	S9	16	0.235307	0.739	18	1.448093	0.266
	S10	16	0.089996	0.848	17	1.381748	0.306
S9	S10	16	0.290716	0.747	17	0.032799	0.941
