# Supplementary material for: Characterization of Microbial Dynamics and Volatile Metabolome Changes During Fermentation of Chambourcin Hybrid Grapes From Two Pennsylvania Regions
Source: Front Microbiol. 2021 Jan 11;11:614278. doi: 10.3389/fmicb.2020.614278 (PMC7829364; doi:10.3389/fmicb.2020.614278)
Supplement: Supplementary file 4 [file Table_4.PDF]

Supplementary Table 4. Selected Chambourcin volatile metabolites detected in samples collected at the last fermentation stage (S10) in all wineries

No.	Compound	RT	RI	No.	Compound	RT	RI
	**Ester**			C37	1-Octanol	11.00	1527
C1	Butanoic acid, methyl ester	3.46	972	C38	2,3-Butanediol, [S-(R*,R*)]-	11.12	1537
C2	Isobutyl acetate	3.81	1001	C39	Propylene Glycol	11.27	1550
C3	Butanoic acid, ethyl ester	4.10	1024	C40	Benzyl alcohol	14.46	1835
C4	Butanoic acid, 3-methyl-, ethyl ester	4.49	1053	C41	Phenylethyl Alcohol	14.89	1875
C5	1-Butanol, 3-methyl-, acetate	5.24	1104		**Acid**		
C6	Acetic acid, pentyl ester	5.88	1150	C42	Acetic acid	9.40	1398
C7	Hexanoic acid, methyl ester	6.06	1163	C43	Propanoic acid, 2-methyl-	10.98	1525
C8	Hexanoic acid, ethyl ester	6.85	1216	C44	Butanoic acid	11.69	1584
C9	Acetic acid, hexyl ester	7.32	1249	C45	Butanoic acid, 3-methyl-	12.19	1628
C10	Ethyl (S)-(-)-lactate	8.18	1308	C46	Butanoic acid, 2-methyl-	12.21	1630
C11	Heptanoic acid, ethyl ester	8.18	1308	C47	Hexanoic acid	14.15	1804
C12	2-Hexenoic acid, ethyl ester	8.28	1316	C48	Heptanoic acid	15.28	1912
C13	Octanoic acid, methyl ester	8.91	1364	C49	(E)-2-Hexenoic acid	15.38	1922
C14	Octanoic acid, ethyl ester	9.63	1417		**Aldehyde**		
C15	Isopentyl hexanoate	9.89	1438	C50	Butanal, 3-methyl-	2.78	906
C16	Pentanoic acid, 2-hydroxy-4-methyl-, ethyl ester	10.80	1510	C51	Hexanal	4.61	1062
C17	Decanoic acid, methyl ester	11.53	1571	C52	Heptanal	6.00	1159

C18	Decanoic acid, ethyl ester	12.13	1623	C53	Benzaldehyde, 4-methyl-	11.96	1607
C19	Octanoic acid, 3-methylbutyl ester	12.35	1642		**Acetal**		
C20	Butanedioic acid, diethyl ester	12.36	1643	C54	Pentane, 1-(1-ethoxyethoxy)-	5.10	1094
C21	Ethyl 9-decenoate	12.64	1668		**Dione**		
C22	Acetic acid, 2-phenylethyl ester	13.89	1781	C55	2,3-Pentanedione	4.31	1040
C23	Dodecanoic acid, ethyl ester	14.39	1828	C56	Acetyl valeryl	5.54	1127
	**Alcohol**			C57	1,2-Cyclopentanedione	13.27	1725
C24	3-Buten-2-ol, 2-methyl-	4.15	1028		**Ketone**		
C25	1-Propanol, 2-methyl-	4.89	1081	C58	2-Heptanone	5.98	1157
C26	1-Butanol	5.52	1125	C59	Acetoin	7.33	1250
C27	1-Butanol, 3-methyl-	6.56	1194	C60	2-Buten-1-one, 1-(2,6,6-trimethyl-1,3-cyclohexadien-1-yl)-, (E)-	14.02	1792
C28	1-Pentanol, 4-methyl-	7.85	1285		**Ether**		
C29	1-Pentanol, 3-methyl-	8.02	1296	C61	1-Propanol, 3-ethoxy-	8.64	1344
C30	1-Hexanol	8.46	1330		**Terpene**		
C31	3-Hexen-1-ol, (Z)-	8.76	1353	C62	D-Limonene	6.19	1171
C32	2-Hexen-1-ol, (Z)-	9.16	1381		**Sulfur**		
C33	1-Hexanol, 2-ethyl-	10.17	1460	C63	1-Propanol, 3-(methylthio)-	12.76	1678
C34	cis-Hept-4-enol	10.30	1470		**Others**		
C35	2,3-Butanediol, [R-(R*,R*)]-	10.70	1501	C64	Benzocyclobutene	6.94	1222
C36	Linalool	10.87	1516				
