# Supplementary material for: Characterization of Microbial Dynamics and Volatile Metabolome Changes During Fermentation of Chambourcin Hybrid Grapes From Two Pennsylvania Regions
Source: Front Microbiol. 2021 Jan 11;11:614278. doi: 10.3389/fmicb.2020.614278 (PMC7829364; doi:10.3389/fmicb.2020.614278)
Supplement: Supplementary file 5 [file Table_5.PDF]

Supplementary Table 5. Loading values in principle component (PC) 1 and 2 of PLS-DA model of volatile metabolites.

	PC1	PC2
Compound No.	Loading values	
C51	0.279	-0.022
C49	0.233	0.187
C1	0.222	-0.061
C52	0.209	0.199
C32	0.164	-0.024
C31	0.122	-0.022
C24	0.115	-0.079
C30	0.097	0.014
C50	0.092	0.013
C48	0.070	0.236
C59	0.053	-0.021
C6	0.011	0.092
C58	-0.012	0.049
C62	-0.026	0.056
C33	-0.037	-0.004
C56	-0.039	0.162
C40	-0.044	0.216
C26	-0.048	0.105
C60	-0.051	-0.006
C53	-0.060	0.037
C43	-0.083	0.160
C7	-0.093	0.223
C47	-0.098	0.204
C45	-0.098	0.166
C42	-0.101	0.135
C57	-0.105	0.010
C34	-0.113	0.209
C46	-0.120	0.152
C25	-0.121	0.093
C36	-0.121	0.085
C27	-0.127	0.098
C9	-0.129	0.100
C12	-0.130	0.214
C2	-0.152	0.101
C28	-0.154	0.127
C55	-0.160	0.070

C44	-0.168	0.092
C4	-0.176	0.123
C3	-0.177	0.108
C29	-0.183	0.092
C37	-0.185	0.014
C61	-0.205	0.121
C5	-0.208	0.103
C19	-0.224	0.144
C16	-0.224	0.168
C64	-0.224	0.162
C8	-0.226	0.213
C38	-0.240	0.206
C21	-0.240	0.102
C63	-0.241	0.169
C15	-0.241	0.096
C41	-0.251	0.217
C22	-0.252	0.073
C54	-0.259	0.180
C14	-0.274	0.159
C13	-0.274	0.104
C23	-0.282	0.133
C11	-0.289	0.169
C17	-0.293	0.096
C35	-0.297	0.180
C39	-0.312	0.152
C10	-0.321	0.089
C20	-0.331	0.145
C18	-0.354	0.171
