# Supplementary material for: Characterization of Microbial Dynamics and Volatile Metabolome Changes During Fermentation of Chambourcin Hybrid Grapes From Two Pennsylvania Regions
Source: Front Microbiol. 2021 Jan 11;11:614278. doi: 10.3389/fmicb.2020.614278 (PMC7829364; doi:10.3389/fmicb.2020.614278)
Supplement: Supplementary file 6 [file Table_6.PDF]

Supplementary Table 6. Averaged relative concentrations of 64 volatile compounds across fermentation stages in Central and East Pennsylvania regions. SD, standard deviation. S, Stages of fermentation

64 core volatile compounds	Central region																			
	Average relative concentrations										SD									
	S1	S2	S3	S4	S5	S6	S7	S8	S9	S10	S1	S2	S3	S4	S5	S6	S7	S8	S9	S10
Butanal, 3-methyl-	0.223	0.168	0.136	0.120	0.093	0.093	0.050	0.029	0.026	0.023	0.252	0.124	0.064	0.088	0.044	0.040	0.039	0.009	0.006	0.007
Butanoic acid, methyl ester	0.142	0.159	0.094	0.070	0.050	0.017	0.000	0.000	0.000	0.000	0.087	0.116	0.103	0.103	0.081	0.038	0.000	0.000	0.000	0.000
Isobutyl acetate	0.104	0.108	0.373	0.417	0.596	0.734	0.789	0.751	0.724	0.711	0.158	0.102	0.282	0.456	0.595	0.689	0.428	0.438	0.403	0.320
Butanoic acid, ethyl ester	0.020	0.063	0.287	0.631	1.122	1.471	1.167	1.496	1.919	1.807	0.017	0.038	0.253	0.745	1.078	1.239	0.730	1.142	1.651	1.535
3-Buten-2-ol, 2-methyl-	0.270	0.394	0.253	0.223	0.164	0.056	0.016	0.020	0.027	0.028	0.306	0.612	0.372	0.314	0.255	0.070	0.013	0.015	0.022	0.018
2,3-Pentanedione	0.025	0.126	0.379	0.347	0.471	0.371	0.256	0.232	0.234	0.203	0.027	0.169	0.271	0.300	0.373	0.216	0.120	0.142	0.144	0.129
Butanoic acid, 3-methyl-, ethyl ester	0.004	0.023	0.026	0.045	0.068	0.086	0.070	0.087	0.109	0.120	0.005	0.026	0.014	0.034	0.034	0.069	0.038	0.041	0.043	0.049
Hexanal	3.695	2.445	1.766	2.219	0.937	0.895	0.026	0.017	0.011	0.007	7.260	4.309	1.828	2.838	1.819	1.855	0.057	0.031	0.018	0.011
1-Propanol, 2-methyl-	1.232	2.086	3.890	4.656	7.214	7.746	6.517	6.169	6.712	6.647	1.353	1.068	1.746	2.713	4.399	5.269	1.888	1.399	1.492	1.572
Pentane, 1-(1-ethoxyethoxy)-	0.001	0.008	0.087	0.158	0.364	0.418	0.476	0.198	0.134	0.120	0.001	0.016	0.114	0.129	0.458	0.341	0.576	0.188	0.120	0.153

1-Butanol, 3-methyl-, acetate	0.597	1.184	6.331	12.237	16.321	21.501	21.253	24.086	24.243	24.211	0.770	0.961	6.530	13.697	18.830	21.867	5.721	9.003	9.558	8.288
1-Butanol	0.038	0.095	0.082	0.100	0.128	0.171	0.161	0.158	0.182	0.191	0.035	0.065	0.056	0.045	0.089	0.126	0.094	0.094	0.078	0.099
Acetyl valeryl	0.010	0.025	0.134	0.144	0.137	0.079	0.090	0.054	0.060	0.051	0.016	0.036	0.202	0.160	0.163	0.057	0.063	0.034	0.026	0.030
Acetic acid, pentyl ester	0.004	0.007	0.015	0.014	0.010	0.011	0.013	0.020	0.014	0.012	0.006	0.006	0.008	0.009	0.004	0.011	0.017	0.026	0.019	0.016
2-Heptanone	0.014	0.012	0.019	0.016	0.041	0.026	0.020	0.034	0.027	0.019	0.016	0.007	0.013	0.007	0.070	0.033	0.021	0.027	0.029	0.021
Heptanal	0.021	0.029	0.027	0.017	0.012	0.011	0.006	0.006	0.006	0.005	0.026	0.043	0.021	0.018	0.014	0.017	0.008	0.005	0.007	0.007
Hexanoic acid, methyl ester	0.021	0.044	0.120	0.130	0.157	0.132	0.143	0.129	0.130	0.128	0.021	0.026	0.096	0.080	0.069	0.054	0.072	0.056	0.035	0.028
D-Limonene	0.000	0.000	0.000	0.000	0.000	0.000	0.000	0.000	0.008	0.000	0.000	0.000	0.000	0.000	0.000	0.000	0.000	0.000	0.019	0.000
1-Butanol, 3-methyl-	12.482	14.793	31.771	47.644	79.798	89.131	84.106	82.020	90.733	90.076	7.131	5.645	12.522	29.368	54.778	63.519	42.165	32.727	34.929	40.475
Hexanoic acid, ethyl ester	0.066	0.216	5.643	9.546	17.224	16.811	21.774	23.743	23.482	21.925	0.131	0.183	9.517	14.598	15.248	13.532	10.502	18.050	15.268	10.942
Benzocyclo butene	0.004	0.285	0.816	0.675	0.720	0.651	0.358	0.333	0.334	0.340	0.005	0.410	0.929	0.677	0.504	0.593	0.217	0.138	0.114	0.104
Acetic acid, hexyl ester	0.121	0.254	0.724	0.864	0.889	0.780	1.629	1.835	1.480	1.397	0.111	0.257	0.599	0.777	0.777	0.320	1.487	1.607	1.450	1.230
Acetoin	0.183	0.222	0.431	0.271	0.134	0.175	0.080	0.036	0.048	0.060	0.261	0.173	0.265	0.329	0.092	0.191	0.139	0.046	0.060	0.066
1-Pentanol, 4-methyl-	0.006	0.017	0.055	0.084	0.113	0.151	0.174	0.169	0.189	0.186	0.003	0.011	0.063	0.104	0.134	0.148	0.109	0.096	0.109	0.114

1-Pentanol, 3-methyl-	0.014	0.029	0.074	0.153	0.218	0.326	0.344	0.340	0.387	0.379	0.014	0.031	0.080	0.215	0.315	0.320	0.253	0.213	0.206	0.217
Ethyl (S)-(-)-lactate	0.002	0.003	0.021	0.034	0.276	0.440	0.629	1.195	2.108	2.554	0.003	0.003	0.017	0.048	0.372	0.559	0.491	0.489	0.485	0.550
Heptanoic acid, ethyl ester	0.008	0.003	0.040	0.068	0.502	0.562	0.733	1.000	1.127	1.411	0.018	0.003	0.028	0.078	0.596	0.504	0.858	1.431	1.713	2.426
2-Hexenoic acid, ethyl ester	0.012	0.015	0.032	0.071	0.161	0.198	0.229	0.254	0.264	0.281	0.009	0.012	0.020	0.046	0.096	0.155	0.128	0.151	0.130	0.165
1-Hexanol	15.130	21.373	23.242	17.572	13.772	13.613	6.799	6.389	6.686	6.757	6.361	16.215	19.478	11.020	9.398	11.156	3.092	3.165	3.288	3.630
1-Propanol, 3-ethoxy-	0.027	0.020	0.051	0.054	0.197	0.126	0.086	0.094	0.120	0.117	0.037	0.043	0.056	0.052	0.336	0.160	0.090	0.082	0.117	0.114
3-Hexen-1-ol, (Z)-	0.895	1.973	1.798	1.321	0.754	0.617	0.217	0.200	0.219	0.218	0.428	1.723	1.873	1.191	0.794	0.644	0.088	0.110	0.127	0.128
Octanoic acid, methyl ester	0.005	0.023	0.174	0.240	0.363	0.381	0.438	0.407	0.421	0.420	0.008	0.030	0.226	0.281	0.321	0.364	0.217	0.246	0.202	0.208
2-Hexen-1-ol, (Z)-	0.458	0.921	0.690	0.658	0.426	0.305	0.059	0.044	0.052	0.054	0.337	1.272	0.944	1.020	0.771	0.535	0.055	0.043	0.046	0.051
Acetic acid	2.896	2.226	2.793	1.764	3.043	3.195	1.945	1.930	2.520	2.963	4.136	1.427	2.332	0.724	2.301	2.226	1.834	1.306	1.462	1.734
Octanoic acid, ethyl ester	0.070	0.429	8.894	18.860	42.339	42.567	43.429	46.225	52.670	47.311	0.065	0.840	15.813	32.571	45.571	46.190	25.361	38.592	43.068	32.816
Isopentyl hexanoate	0.005	0.009	0.076	0.148	0.292	0.315	0.265	0.249	0.261	0.233	0.011	0.017	0.145	0.280	0.376	0.378	0.201	0.174	0.131	0.099
1-Hexanol, 2-ethyl-	0.146	0.113	0.121	0.124	0.156	0.155	0.144	0.185	0.147	0.145	0.041	0.028	0.046	0.054	0.085	0.062	0.043	0.101	0.043	0.044
cis-Hept-4-enol	0.003	0.013	0.026	0.026	0.032	0.038	0.036	0.034	0.038	0.037	0.003	0.014	0.018	0.016	0.021	0.022	0.019	0.019	0.019	0.017

2,3-Butanediol, [R-(R*,R*)]-	0.041	0.029	0.320	0.472	1.694	1.970	1.936	2.476	2.659	2.909	0.081	0.035	0.360	0.545	1.898	1.752	0.616	0.799	0.961	1.070
Pentanoic acid, 2-hydroxy-4-methyl-, ethyl ester	0.002	0.001	0.004	0.006	0.024	0.037	0.063	0.103	0.147	0.191	0.002	0.002	0.003	0.006	0.026	0.050	0.043	0.031	0.041	0.061
Linalool	0.023	0.031	0.060	0.051	0.073	0.088	0.115	0.122	0.131	0.132	0.019	0.015	0.043	0.045	0.053	0.060	0.039	0.042	0.037	0.033
Propanoic acid, 2-methyl-	0.179	0.494	0.438	0.550	0.627	0.646	0.414	0.407	0.433	0.427	0.251	0.627	0.131	0.113	0.245	0.504	0.185	0.229	0.251	0.268
1-Octanol	0.074	0.093	0.243	0.206	0.331	0.383	0.478	0.498	0.535	0.567	0.092	0.082	0.270	0.258	0.301	0.325	0.191	0.188	0.181	0.177
2,3-Butanediol, [S-(R*,R*)]-	0.039	0.016	0.080	0.092	0.484	0.454	0.491	0.701	0.855	0.971	0.079	0.024	0.070	0.103	0.697	0.428	0.226	0.341	0.453	0.523
Propylene Glycol	0.001	0.001	0.003	0.010	0.052	0.075	0.058	0.078	0.098	0.106	0.002	0.002	0.002	0.011	0.059	0.073	0.018	0.019	0.026	0.038
Decanoic acid, methyl ester	0.000	0.002	0.082	0.074	0.215	0.167	0.152	0.142	0.166	0.149	0.001	0.003	0.140	0.115	0.226	0.179	0.093	0.118	0.116	0.098
Butanoic acid	0.011	0.040	0.107	0.133	0.185	0.198	0.180	0.196	0.215	0.209	0.013	0.033	0.091	0.105	0.138	0.132	0.067	0.083	0.098	0.072
Benzaldehyde, 4-methyl-	0.148	0.143	0.239	0.221	0.338	0.278	0.301	0.284	0.330	0.355	0.046	0.085	0.073	0.120	0.281	0.158	0.133	0.148	0.155	0.165
Decanoic acid, ethyl ester	0.037	0.112	2.948	7.800	31.012	22.833	18.070	21.349	28.469	25.442	0.063	0.133	4.972	13.215	36.028	24.792	12.098	20.275	27.093	21.419
Butanoic acid, 3-methyl-	0.127	0.354	0.387	0.489	0.548	0.576	0.445	0.439	0.466	0.479	0.158	0.421	0.233	0.216	0.323	0.518	0.175	0.191	0.232	0.245
Butanoic acid, 2-methyl-	0.054	0.200	0.198	0.250	0.307	0.288	0.275	0.254	0.271	0.275	0.060	0.219	0.114	0.118	0.148	0.166	0.139	0.112	0.135	0.142
Octanoic acid, 3-methylbutyl ester	0.004	0.005	0.102	0.230	0.503	0.588	0.497	0.507	0.614	0.548	0.008	0.008	0.195	0.411	0.623	0.735	0.373	0.488	0.521	0.365

Butanedioic acid, diethyl ester	0.001	0.001	0.024	0.045	0.346	0.209	0.168	0.276	0.550	0.619	0.001	0.002	0.042	0.081	0.571	0.200	0.173	0.039	0.361	0.256
Ethyl 9-decenoate	0.003	0.005	0.047	0.139	1.098	1.110	0.659	0.482	0.397	0.450	0.002	0.005	0.076	0.247	1.674	1.564	0.385	0.324	0.262	0.332
1-Propanol, 3-(methylthio)-	0.032	0.249	0.507	0.490	0.672	0.799	0.558	0.530	0.603	0.671	0.043	0.473	0.549	0.580	0.643	0.748	0.344	0.208	0.272	0.328
1,2-Cyclopentanedione	0.170	0.198	0.248	0.172	1.042	0.995	0.518	1.426	0.941	0.521	0.180	0.113	0.213	0.087	1.713	1.333	0.596	1.644	1.281	0.356
Acetic acid, 2-phenylethyl ester	0.096	0.117	0.440	0.740	1.079	1.339	1.859	1.975	1.834	1.849	0.146	0.138	0.293	0.837	1.097	1.207	0.738	0.883	0.739	0.849
2-Buten-1-one, 1-(2,6,6-trimethyl-1,3-cyclohexadien-1-yl)-, (E)-	0.078	0.075	0.090	0.098	0.091	0.082	0.121	0.140	0.146	0.162	0.030	0.019	0.026	0.030	0.026	0.034	0.032	0.043	0.044	0.038
Hexanoic acid	0.193	0.337	1.077	1.362	2.054	2.033	2.168	2.447	2.581	2.606	0.173	0.246	1.107	1.272	1.413	1.391	0.919	1.231	1.334	0.970
Dodecanoic acid, ethyl ester	0.004	0.005	0.690	1.535	6.824	3.670	2.956	4.053	5.320	4.583	0.010	0.006	1.186	2.626	9.375	3.941	2.190	5.264	6.699	5.494
Benzyl alcohol	0.007	0.032	0.052	0.036	0.080	0.072	0.050	0.056	0.060	0.072	0.011	0.023	0.014	0.017	0.082	0.042	0.027	0.022	0.015	0.026
Phenylethyl Alcohol	0.560	0.895	4.296	6.940	19.795	21.664	21.849	23.211	25.147	26.619	1.021	0.947	4.295	8.665	18.531	18.808	11.766	11.766	12.449	13.890
Heptanoic acid	0.012	0.010	0.022	0.019	0.047	0.053	0.028	0.038	0.035	0.035	0.019	0.009	0.012	0.009	0.076	0.048	0.013	0.025	0.017	0.018
(E)-2-Hexenoic acid	0.109	0.076	0.099	0.094	0.097	0.063	0.025	0.027	0.024	0.025	0.062	0.032	0.044	0.082	0.086	0.078	0.029	0.035	0.030	0.031
64 core volatile compounds	East region																			

	Average relative concentrations										SD									
	S1	S2	S3	S4	S5	S6	S7	S8	S9	S10	S1	S2	S3	S4	S5	S6	S7	S8	S9	S10
Butanal, 3-methyl-	0.054	0.101	0.102	0.095	0.083	0.054	0.039	0.027	0.027	0.023	0.021	0.063	0.058	0.060	0.035	0.028	0.017	0.011	0.009	0.006
Butanoic acid, methyl ester	0.131	0.116	0.086	0.041	0.012	0.020	0.013	0.016	0.020	0.024	0.116	0.119	0.076	0.036	0.013	0.021	0.014	0.011	0.014	0.021
Isobutyl acetate	0.030	0.071	0.179	0.235	0.380	0.741	0.638	0.593	0.523	0.523	0.025	0.030	0.049	0.073	0.076	0.408	0.456	0.435	0.237	0.239
Butanoic acid, ethyl ester	0.026	0.028	0.045	0.073	0.310	1.072	0.948	0.840	0.864	0.893	0.038	0.034	0.035	0.058	0.362	0.534	0.435	0.338	0.271	0.238
3-Buten-2-ol, 2-methyl-	0.316	0.256	0.128	0.058	0.035	0.034	0.033	0.033	0.038	0.038	0.262	0.220	0.112	0.055	0.023	0.023	0.014	0.014	0.018	0.018
2,3-Pentanedione	0.005	0.078	0.244	0.254	0.318	0.356	0.285	0.250	0.220	0.214	0.001	0.081	0.196	0.168	0.152	0.096	0.141	0.121	0.098	0.109
Butanoic acid, 3-methyl-, ethyl ester	0.000	0.004	0.011	0.013	0.023	0.061	0.081	0.098	0.120	0.138	0.001	0.004	0.013	0.011	0.019	0.034	0.028	0.040	0.045	0.062
Hexanal	0.431	0.226	0.321	0.154	0.008	0.019	0.001	0.001	0.001	0.001	0.516	0.342	0.539	0.263	0.013	0.037	0.002	0.002	0.001	0.002
1-Propanol, 2-methyl-	0.451	1.465	2.566	2.975	3.496	7.679	7.044	6.687	6.753	6.763	0.315	0.545	1.261	1.439	1.482	2.666	3.167	2.776	2.565	2.648
Pentane, 1-(1-ethoxyethoxy)-	0.000	0.000	0.003	0.070	0.185	0.460	0.266	0.134	0.086	0.062	0.000	0.000	0.006	0.104	0.162	0.068	0.161	0.096	0.072	0.055
1-Butanol, 3-methyl-, acetate	0.078	0.217	1.417	4.938	10.021	27.196	21.796	20.113	19.593	19.856	0.059	0.132	0.352	3.407	1.671	9.634	7.240	6.285	5.409	4.223
1-Butanol	0.032	0.051	0.060	0.039	0.037	0.112	0.117	0.111	0.116	0.111	0.022	0.037	0.047	0.021	0.021	0.038	0.031	0.013	0.012	0.014

Acetyl valeryl	0.003	0.006	0.042	0.214	0.328	0.107	0.078	0.056	0.046	0.034	0.003	0.006	0.035	0.277	0.273	0.082	0.071	0.046	0.042	0.029
Acetic acid, pentyl ester	0.001	0.011	0.006	0.009	0.015	0.017	0.013	0.011	0.012	0.011	0.002	0.014	0.005	0.011	0.019	0.013	0.008	0.008	0.010	0.009
2-Heptanone	0.005	0.046	0.044	0.061	0.077	0.053	0.043	0.038	0.035	0.031	0.004	0.053	0.046	0.055	0.093	0.062	0.049	0.045	0.041	0.042
Heptanal	0.001	0.013	0.010	0.004	0.002	0.001	0.001	0.001	0.000	0.000	0.003	0.012	0.010	0.007	0.004	0.002	0.001	0.001	0.001	0.001
Hexanoic acid, methyl ester	0.001	0.010	0.032	0.042	0.115	0.123	0.078	0.072	0.080	0.085	0.002	0.009	0.032	0.027	0.140	0.081	0.038	0.028	0.027	0.031
D-Limonene	0.000	0.000	0.000	0.000	0.000	0.000	0.000	0.194	0.148	0.185	0.000	0.000	0.000	0.000	0.000	0.000	0.000	0.389	0.297	0.369
1-Butanol, 3-methyl-	3.287	11.558	22.020	28.469	40.603	90.325	82.037	76.819	81.874	81.389	2.647	2.381	9.950	20.460	24.365	19.050	21.372	20.533	17.859	22.137
Hexanoic acid, ethyl ester	0.009	0.066	0.452	2.364	9.571	25.128	16.827	14.387	14.467	15.074	0.016	0.102	0.524	2.528	11.565	15.591	10.376	5.990	4.543	5.435
Benzocyclo butene	0.029	0.055	0.109	0.150	0.202	0.468	0.280	0.233	0.255	0.269	0.050	0.085	0.121	0.062	0.058	0.271	0.150	0.176	0.203	0.156
Acetic acid, hexyl ester	0.055	0.168	0.405	0.837	1.363	2.520	1.553	1.271	1.134	1.034	0.068	0.204	0.204	0.417	0.273	1.226	0.717	0.533	0.694	0.537
Acetoin	0.044	0.132	0.318	0.311	0.400	0.032	0.033	0.029	0.036	0.036	0.051	0.084	0.152	0.370	0.470	0.025	0.022	0.019	0.024	0.033
1-Pentanol, 4-methyl-	0.003	0.007	0.024	0.060	0.097	0.361	0.275	0.254	0.276	0.253	0.003	0.004	0.008	0.049	0.057	0.314	0.173	0.139	0.163	0.122
1-Pentanol, 3-methyl-	0.002	0.009	0.036	0.061	0.090	0.352	0.320	0.307	0.321	0.302	0.001	0.003	0.023	0.057	0.063	0.292	0.262	0.219	0.185	0.174
Ethyl (S)-(-)-lactate	0.002	0.000	0.035	0.073	0.092	0.235	0.323	0.604	0.817	1.088	0.004	0.000	0.058	0.115	0.113	0.166	0.324	0.889	1.237	1.684

Heptanoic acid, ethyl ester	0.000	0.001	0.005	0.008	0.028	0.312	0.214	0.191	0.160	0.152	0.000	0.001	0.008	0.009	0.039	0.173	0.141	0.126	0.082	0.063
2-Hexenoic acid, ethyl ester	0.000	0.002	0.003	0.003	0.013	0.153	0.139	0.139	0.154	0.161	0.001	0.002	0.004	0.002	0.013	0.127	0.092	0.072	0.100	0.095
1-Hexanol	13.434	21.145	21.712	14.972	13.181	7.582	6.576	6.139	6.009	5.774	6.045	10.852	8.899	9.207	9.482	2.775	1.591	1.243	1.369	1.122
1-Propanol, 3-ethoxy-	0.005	0.017	0.043	0.038	0.134	0.151	0.126	0.104	0.097	0.093	0.007	0.015	0.040	0.042	0.193	0.194	0.166	0.130	0.121	0.117
3-Hexen-1-ol, (Z)-	0.792	0.915	0.824	0.484	0.381	0.203	0.166	0.163	0.168	0.161	0.313	0.289	0.199	0.298	0.311	0.131	0.074	0.075	0.086	0.078
Octanoic acid, methyl ester	0.000	0.001	0.020	0.070	0.221	0.422	0.281	0.235	0.219	0.221	0.001	0.002	0.027	0.061	0.295	0.256	0.186	0.082	0.063	0.063
2-Hexen-1-ol, (Z)-	0.486	0.517	0.519	0.261	0.176	0.049	0.033	0.031	0.027	0.027	0.246	0.232	0.268	0.154	0.105	0.018	0.010	0.009	0.009	0.010
Acetic acid	0.579	1.343	2.136	1.701	1.646	3.168	3.160	3.271	3.338	3.944	1.002	1.109	0.646	0.913	1.108	1.784	2.314	2.569	2.791	3.712
Octanoic acid, ethyl ester	0.006	0.010	0.486	3.487	13.250	51.830	34.435	27.123	20.710	19.943	0.010	0.008	0.492	3.780	16.735	31.745	25.288	13.138	10.785	8.471
Isopentyl hexanoate	0.001	0.002	0.003	0.026	0.052	0.305	0.169	0.121	0.096	0.092	0.001	0.000	0.000	0.039	0.044	0.219	0.147	0.057	0.047	0.036
1-Hexanol, 2-ethyl-	0.140	0.125	0.126	0.125	0.127	0.127	0.139	0.143	0.143	0.139	0.012	0.013	0.023	0.020	0.007	0.027	0.020	0.017	0.015	0.014
cis-Hept-4-enol	0.001	0.006	0.021	0.025	0.031	0.047	0.053	0.052	0.051	0.052	0.002	0.006	0.012	0.010	0.019	0.028	0.029	0.024	0.020	0.019
2,3-Butanediol, [R-(R*,R*)]-	0.002	0.017	0.075	0.245	0.694	3.638	2.346	2.363	2.460	2.004	0.004	0.015	0.090	0.273	0.741	2.619	1.341	1.012	0.891	0.550
Pentanoic acid, 2-hydroxy-4-methyl-, ethyl ester	0.001	0.002	0.002	0.003	0.012	0.034	0.077	0.118	0.162	0.197	0.002	0.003	0.003	0.002	0.012	0.026	0.034	0.056	0.057	0.060

Linalool	0.007	0.014	0.023	0.052	0.066	0.099	0.110	0.104	0.097	0.102	0.006	0.010	0.006	0.033	0.031	0.017	0.015	0.025	0.028	0.026
Propanoic acid, 2-methyl-	0.036	0.333	0.447	0.259	0.284	0.443	0.399	0.388	0.356	0.354	0.058	0.269	0.390	0.072	0.054	0.202	0.261	0.206	0.161	0.160
1-Octanol	0.029	0.395	0.563	0.702	0.948	0.955	0.953	1.087	1.060	1.113	0.039	0.649	0.847	0.956	0.747	0.963	0.848	1.047	1.041	1.092
2,3-Butanediol, [S-(R*,R*)]-	0.006	0.011	0.016	0.044	0.103	0.988	0.615	0.712	0.796	0.722	0.010	0.015	0.014	0.048	0.071	0.628	0.299	0.254	0.238	0.438
Propylene Glycol	0.000	0.000	0.000	0.000	0.006	0.126	0.072	0.079	0.091	0.073	0.000	0.000	0.000	0.000	0.011	0.103	0.041	0.029	0.035	0.032
Decanoic acid, methyl ester	0.001	0.002	0.007	0.018	0.081	0.240	0.149	0.111	0.088	0.078	0.001	0.001	0.004	0.011	0.109	0.115	0.107	0.074	0.070	0.060
Butanoic acid	0.002	0.017	0.035	0.039	0.073	0.124	0.128	0.133	0.144	0.153	0.001	0.006	0.023	0.027	0.052	0.070	0.034	0.061	0.077	0.085
Benzaldehyde, 4-methyl-	0.119	0.155	0.204	0.225	0.261	0.301	0.306	0.304	0.279	0.261	0.037	0.023	0.046	0.040	0.081	0.110	0.075	0.091	0.040	0.066
Decanoic acid, ethyl ester	0.000	0.030	0.160	0.867	5.072	24.886	16.740	12.808	8.878	7.964	0.000	0.053	0.226	0.853	7.083	15.243	13.642	8.683	7.885	6.895
Butanoic acid, 3-methyl-	0.031	0.267	0.336	0.270	0.389	0.635	0.542	0.534	0.525	0.529	0.054	0.247	0.330	0.090	0.169	0.330	0.308	0.283	0.249	0.253
Butanoic acid, 2-methyl-	0.005	0.077	0.134	0.199	0.256	0.443	0.365	0.379	0.374	0.383	0.009	0.044	0.090	0.195	0.173	0.164	0.187	0.224	0.223	0.245
Octanoic acid, 3-methylbutyl ester	0.001	0.001	0.001	0.047	0.129	0.585	0.369	0.271	0.192	0.163	0.000	0.001	0.001	0.074	0.130	0.472	0.347	0.165	0.158	0.142
Butanedioic acid, diethyl ester	0.000	0.000	0.000	0.004	0.016	0.179	0.209	0.276	0.361	0.430	0.001	0.000	0.000	0.006	0.020	0.083	0.115	0.139	0.208	0.192
Ethyl 9-decenoate	0.000	0.001	0.004	0.017	0.045	0.361	0.295	0.230	0.127	0.110	0.001	0.001	0.004	0.018	0.043	0.395	0.397	0.292	0.070	0.062

1-Propanol, 3-(methylthio)-	0.000	0.012	0.106	0.184	0.274	0.683	0.549	0.475	0.502	0.489	0.000	0.018	0.025	0.060	0.051	0.224	0.188	0.176	0.207	0.142
1,2-Cyclopentanedione	0.398	0.224	0.329	0.247	0.100	1.365	1.371	1.190	1.044	0.989	0.521	0.041	0.121	0.105	0.012	0.856	1.150	0.728	0.777	0.390
Acetic acid, 2-phenylethyl ester	0.008	0.018	0.132	1.006	1.900	3.737	2.863	2.690	2.096	2.018	0.008	0.018	0.108	1.116	1.241	1.895	2.054	1.696	1.139	1.070
2-Buten-1-one, 1-(2,6,6-trimethyl-1,3-cyclohexadien-1-yl)-, (E)-	0.086	0.096	0.091	0.107	0.123	0.078	0.108	0.125	0.140	0.146	0.056	0.068	0.058	0.034	0.022	0.012	0.043	0.056	0.059	0.064
Hexanoic acid	0.027	0.159	0.369	0.450	1.050	1.430	1.306	1.278	1.337	1.375	0.024	0.163	0.347	0.265	1.262	0.873	0.705	0.596	0.508	0.528
Dodecanoic acid, ethyl ester	0.007	0.010	0.026	0.147	0.768	3.647	2.367	1.821	1.314	1.199	0.006	0.006	0.014	0.173	1.029	1.858	1.767	1.336	1.124	1.116
Benzyl alcohol	0.003	0.012	0.019	0.018	0.024	0.043	0.043	0.040	0.039	0.041	0.004	0.011	0.016	0.015	0.021	0.020	0.020	0.020	0.016	0.013
Phenylethyl Alcohol	0.025	0.120	1.184	8.240	11.796	39.496	35.404	32.381	32.152	33.001	0.043	0.163	0.597	12.348	10.455	13.023	11.608	11.984	10.221	13.604
Heptanoic acid	0.003	0.010	0.011	0.012	0.013	0.011	0.011	0.011	0.010	0.012	0.003	0.009	0.010	0.011	0.012	0.013	0.011	0.013	0.012	0.014
(E)-2-Hexenoic acid	0.014	0.035	0.021	0.011	0.009	0.000	0.000	0.000	0.001	0.001	0.018	0.043	0.019	0.010	0.011	0.000	0.000	0.000	0.002	0.003
