# Supplementary material for: Characterization of Microbial Dynamics and Volatile Metabolome Changes During Fermentation of Chambourcin Hybrid Grapes From Two Pennsylvania Regions
Source: Front Microbiol. 2021 Jan 11;11:614278. doi: 10.3389/fmicb.2020.614278 (PMC7829364; doi:10.3389/fmicb.2020.614278)
Supplement: Supplementary file 7 [file Table_7.PDF]

Supplementary Table 7. Significant differentially volatile compounds between each winery in the Central or East region across fermentation stages as calculated by Kruskal–Wallis Test (one-way ANOVA on ranks), indicated by the FDR-adjusted *p*-value. FDR-adjusted *p*-values > 0.05 remained blank in the table. S, Stages of fermentation.

64 core volatile compounds	Central region										East region									
	S1	S2	S3	S4	S5	S6	S7	S8	S9	S10	S1	S2	S3	S4	S5	S6	S7	S8	S9	S10
(E)-2-Hexenoic acid			0.028	0.025	0.028	0.017	0.021		0.022	0.023			0.039		0.040				0.039	0.035
1,2-Cyclopentanedione	0.030						0.025		0.050	0.046										
1-Butanol	0.019	0.020	0.028	0.028	0.028	0.020	0.019		0.022	0.023			0.039	0.036	0.040		0.035		0.039	
1-Butanol, 3-methyl-	0.019	0.020	0.028	0.025	0.028	0.017	0.019	0.036	0.022	0.023			0.039	0.036	0.040	0.048	0.035	0.036	0.039	0.035
1-Butanol, 3-methyl-, acetate	0.019	0.020	0.028	0.025	0.028	0.017	0.019	0.036	0.022	0.023				0.036		0.035	0.035	0.036	0.039	0.036
1-Hexanol	0.019	0.020	0.028	0.025	0.028	0.017	0.019		0.022	0.023			0.039	0.036	0.040	0.035	0.035		0.039	0.035
1-Hexanol, 2-ethyl-	0.019	0.020	0.030	0.028	0.030	0.017	0.019		0.022	0.023			0.039	0.048		0.042	0.046		0.042	
1-Octanol	0.019	0.020	0.028	0.025	0.028	0.020	0.021	0.036	0.022	0.027			0.039	0.036	0.040	0.035	0.035	0.036	0.039	0.035
1-Pentanol, 3-methyl-	0.020	0.020	0.028	0.025	0.028	0.017	0.019	0.036	0.022	0.023			0.039	0.036		0.035	0.035	0.036	0.039	0.035
1-Pentanol, 4-methyl-	0.034	0.020	0.028	0.028	0.028	0.017	0.019	0.036	0.022	0.023			0.039	0.036		0.035	0.035	0.036	0.039	0.035
1-Propanol, 2-methyl-	0.019	0.020	0.030	0.025	0.030	0.017	0.019	0.036	0.022	0.023			0.039	0.048	0.040	0.035	0.035	0.036	0.039	0.035
1-Propanol, 3-(methylthio)-	0.019	0.020	0.028	0.025	0.028	0.017	0.019	0.036	0.022	0.030				0.036			0.035	0.036	0.039	0.035

1-Propanol, 3-ethoxy-	0.019	0.020	0.028	0.025	0.028	0.017	0.020	0.036	0.022	0.023			0.039	0.036	0.040	0.035	0.035		0.039	0.035
2,3-Butanediol, [R-(R*,R*)]-	0.019	0.020	0.039	0.025	0.039	0.031		0.036		0.035				0.036	0.040	0.035	0.035	0.036	0.050	
2,3-Butanediol, [S-(R*,R*)]-	0.019	0.020		0.028		0.020			0.044	0.025			0.048	0.036	0.049	0.035	0.039			0.035
2,3-Pentanedione	0.019	0.020	0.030	0.028	0.030		0.034		0.026	0.033			0.039		0.040		0.035			0.040
2-Buten-1-one, 1-(2,6,6-trimethyl-1,3-cyclohexadien-1-yl)-, (E)-																				0.035
2-Heptanone	0.019		0.042		0.042	0.017	0.019	0.036	0.023	0.023			0.039	0.036	0.040	0.035	0.035	0.036	0.039	0.035
2-Hexen-1-ol, (Z)-	0.019	0.020		0.028	0.028	0.029	0.019	0.036	0.031	0.023			0.039	0.036	0.040	0.035	0.038	0.036	0.039	0.035
2-Hexenoic acid, ethyl ester	0.019	0.020	0.030	0.025	0.030	0.017	0.019	0.036	0.022	0.023			0.039		0.040	0.035	0.035	0.036	0.039	0.035
3-Buten-2-ol, 2-methyl-	0.024	0.020	0.030	0.028	0.030	0.039	0.030	0.045	0.022	0.033			0.039		0.049			0.045	0.044	0.035
3-Hexen-1-ol, (Z)-	0.019	0.020	0.028	0.025	0.028	0.017	0.019	0.036	0.022	0.023			0.039	0.036	0.040	0.035	0.035	0.036	0.039	0.035
Acetic acid	0.019	0.020	0.030	0.025	0.030	0.017	0.020	0.036	0.022	0.026			0.039	0.036	0.040	0.035	0.035	0.036	0.039	0.035
Acetic acid, 2-phenylethyl ester				0.025		0.017	0.020	0.036	0.022	0.023			0.039	0.036	0.040	0.035	0.035	0.036	0.039	0.035
Acetic acid, hexyl ester	0.019	0.020	0.028	0.025	0.028	0.020	0.019	0.036	0.022	0.023			0.039	0.036		0.035	0.035	0.036	0.039	0.035
Acetic acid, pentyl ester	0.040	0.020	0.030		0.030	0.017	0.019	0.036	0.022	0.023				0.036	0.040	0.044	0.035	0.036	0.039	0.035
Acetoin	0.019	0.020	0.028	0.028	0.028	0.017	0.019		0.022	0.023			0.039	0.036			0.048			0.035

Acetyl valeryl	0.019	0.020	0.028	0.025	0.028		0.039	0.036		0.032			0.039	0.036		0.042	0.035	0.036	0.039	0.035
Benzaldehyde, 4-methyl-		0.024					0.019	0.045	0.026	0.023						0.042	0.046	0.045		
Benzocyclobutene	0.019	0.020	0.028	0.025	0.028	0.017	0.019	0.036	0.022	0.023			0.039		0.049	0.035	0.035	0.036	0.039	0.035
Benzyl alcohol		0.020	0.030	0.025	0.030	0.017	0.021	0.049	0.042	0.031				0.036	0.040	0.035	0.035	0.049		0.035
Butanal, 3-methyl-	0.019	0.020	0.030	0.028	0.030	0.020	0.020	0.036	0.027	0.035			0.039	0.036		0.035	0.035	0.036	0.050	
Butanedioic acid, diethyl ester	0.019	0.020	0.030	0.025	0.030	0.017	0.019	0.049	0.022	0.035			0.039	0.036	0.040	0.035	0.035	0.049	0.039	0.035
Butanoic acid	0.020	0.020	0.028	0.025	0.028	0.017	0.019	0.036	0.034	0.023			0.039	0.036	0.040	0.035	0.035	0.036	0.039	0.035
Butanoic acid, 2-methyl-	0.019	0.020	0.030	0.025	0.030	0.017	0.019	0.036	0.027	0.023			0.039	0.036	0.040	0.035	0.035	0.036	0.039	0.035
Butanoic acid, 3-methyl-	0.019	0.020	0.030	0.025	0.030	0.017	0.019	0.036	0.022	0.023			0.039	0.036	0.040		0.035	0.036	0.039	0.035
Butanoic acid, 3-methyl-, ethyl ester	0.019	0.020	0.028	0.028	0.028	0.017	0.034		0.037	0.033			0.039	0.036	0.049					0.040
Butanoic acid, ethyl ester	0.024	0.021	0.028	0.025	0.028	0.017	0.019	0.036	0.022	0.023			0.039	0.036	0.040	0.040	0.035	0.036	0.039	0.035
Butanoic acid, methyl ester	0.022	0.020	0.028	0.025	0.028	0.020	0.019		0.022				0.039	0.036		0.035	0.038		0.039	0.035
cis-Hept-4-enol	0.019	0.020	0.028	0.028	0.028	0.017	0.019	0.036	0.022	0.023			0.039	0.036	0.040	0.035	0.035	0.036		0.035
Decanoic acid, ethyl ester	0.041	0.020	0.028	0.025	0.028	0.017	0.019	0.036	0.022	0.023			0.039	0.036	0.040	0.035	0.035	0.036	0.039	0.035
Decanoic acid, methyl ester			0.028	0.025	0.028	0.017		0.036		0.023					0.040	0.035	0.035	0.036	0.039	0.035

D-Limonene								0.036										0.036	0.039	0.035
Dodecanoic acid, ethyl ester	0.023	0.020	0.030	0.025	0.030	0.017		0.036	0.022	0.023				0.036		0.035	0.035	0.036	0.039	0.035
Ethyl (S)-(-)-lactate	0.019	0.020	0.037	0.025	0.037	0.017	0.019	0.036		0.044			0.039	0.036	0.040	0.047	0.035	0.036		0.035
Ethyl 9-decenoate				0.028	0.030	0.017	0.019	0.036	0.022	0.023			0.039	0.036	0.040	0.035	0.035	0.036	0.039	0.035
Heptanal		0.020	0.046	0.025	0.046	0.017	0.019	0.036	0.022	0.023				0.045	0.040	0.035	0.035	0.036	0.039	
Heptanoic acid	0.024	0.022	0.028	0.028	0.028	0.020		0.038	0.048	0.023					0.040	0.035	0.035	0.038	0.039	0.035
Heptanoic acid, ethyl ester	0.034	0.032	0.046	0.032	0.046	0.017	0.019	0.036	0.022	0.023			0.039	0.036	0.040	0.035	0.035	0.036	0.039	
Hexanal	0.019	0.020	0.030	0.025	0.030	0.017	0.019	0.036	0.022	0.023			0.039	0.036	0.040	0.035	0.035	0.036	0.039	0.035
Hexanoic acid	0.019	0.022	0.028	0.025	0.028	0.017	0.019	0.036	0.031	0.029			0.039	0.036	0.040	0.035	0.035	0.036	0.039	0.035
Hexanoic acid, ethyl ester	0.019	0.020	0.028	0.025	0.028	0.017		0.036	0.022	0.023			0.039	0.036	0.040	0.035	0.035	0.036	0.039	0.035
Hexanoic acid, methyl ester	0.019	0.020	0.028	0.025	0.028	0.017							0.039	0.036	0.040	0.035	0.035			
Isobutyl acetate	0.019	0.020	0.028	0.025	0.028	0.017	0.019	0.036	0.022	0.023				0.036	0.049	0.035	0.035	0.036	0.039	0.035
Isopentyl hexanoate	0.019	0.020	0.028	0.025	0.028	0.017	0.019	0.036	0.022	0.029				0.036	0.040	0.035		0.036	0.039	0.035
Linalool	0.031		0.045		0.045	0.022	0.034	0.036	0.037					0.036	0.049			0.036	0.042	0.042
Octanoic acid, 3-methylbutyl ester	0.020	0.049	0.028	0.025	0.028	0.017	0.019	0.036	0.022	0.023				0.036	0.040	0.035	0.035	0.036	0.039	0.043

Octanoic acid, ethyl ester	0.019	0.020	0.028	0.025	0.028	0.017	0.019	0.036	0.023	0.023			0.039	0.036	0.040	0.035	0.035	0.036	0.039	0.035
Octanoic acid, methyl ester	0.019	0.020	0.028	0.025	0.028	0.017	0.019	0.036	0.022	0.023			0.039	0.036	0.040	0.035	0.035	0.036		
Pentane, 1-(1-ethoxyethoxy)-	0.019	0.020	0.043	0.028	0.043	0.020	0.030						0.048	0.036			0.047		0.048	0.035
Pentanoic acid, 2-hydroxy-4-methyl-, ethyl ester	0.019			0.028		0.020	0.020		0.044	0.031			0.039			0.035			0.039	0.040
Phenylethyl Alcohol	0.019	0.020	0.028	0.028	0.028	0.017	0.019	0.036	0.031	0.035			0.039	0.036	0.040		0.036	0.036	0.047	0.035
Propanoic acid, 2-methyl-	0.019	0.020	0.028		0.028	0.017	0.019	0.036	0.022	0.033			0.039	0.036	0.040	0.035	0.035	0.036	0.039	0.035
Propylene Glycol	0.022	0.020	0.028	0.025	0.028	0.017				0.042					0.049	0.035	0.041			0.040
