# Supplementary material for: Characterization of Microbial Dynamics and Volatile Metabolome Changes During Fermentation of Chambourcin Hybrid Grapes From Two Pennsylvania Regions
Source: Front Microbiol. 2021 Jan 11;11:614278. doi: 10.3389/fmicb.2020.614278 (PMC7829364; doi:10.3389/fmicb.2020.614278)
Supplement: Supplementary file 12 [file Image_1.pdf]

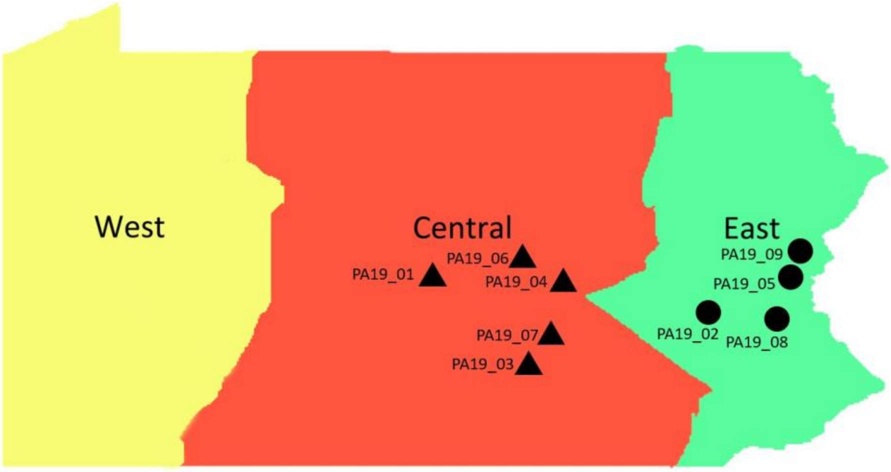

Supplementary Figure 1. Sample collection sites from Central and East Pennsylvania. ▲ or ● represents individual wineries from which Chambourcin samples throughout fermentation processes were collected from the Central or East regions.

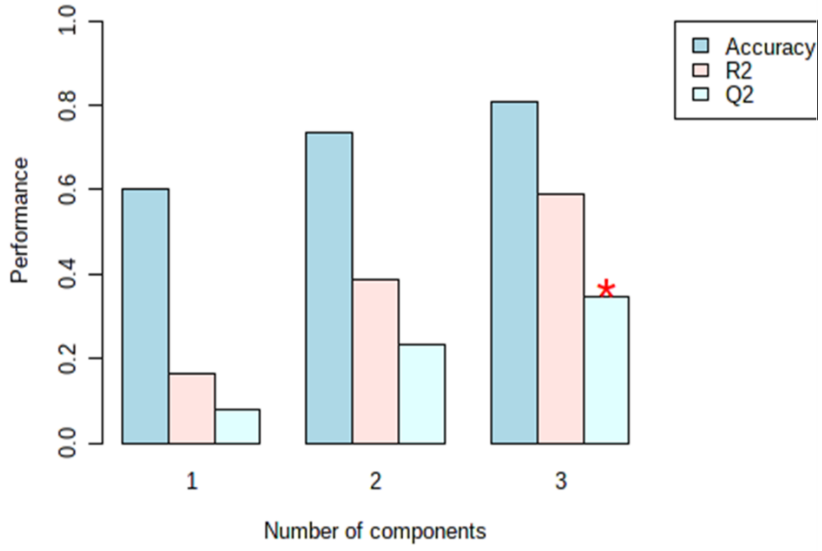

Supplementary Figure 2. The result of PLS-DA model classification using ‘Leave-one-out cross-validation’ approach. 3 components were shown cumulatively.

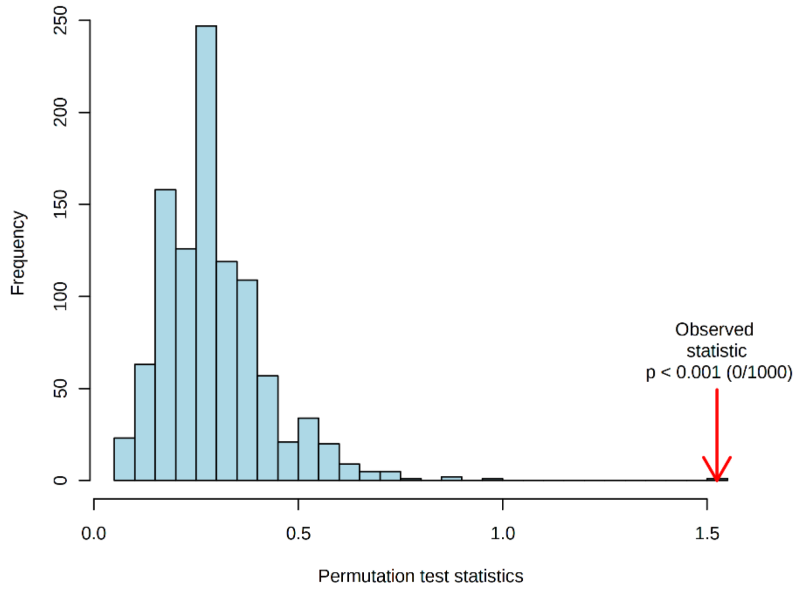

Supplementary Figure 3. PLS-DA model validation by permutation tests based on the ratio of the between group sum of the squares and the within group sum of squares (B/W-ratio). The p-value based on permutation is $p < 0.001$ (0/1000).

A

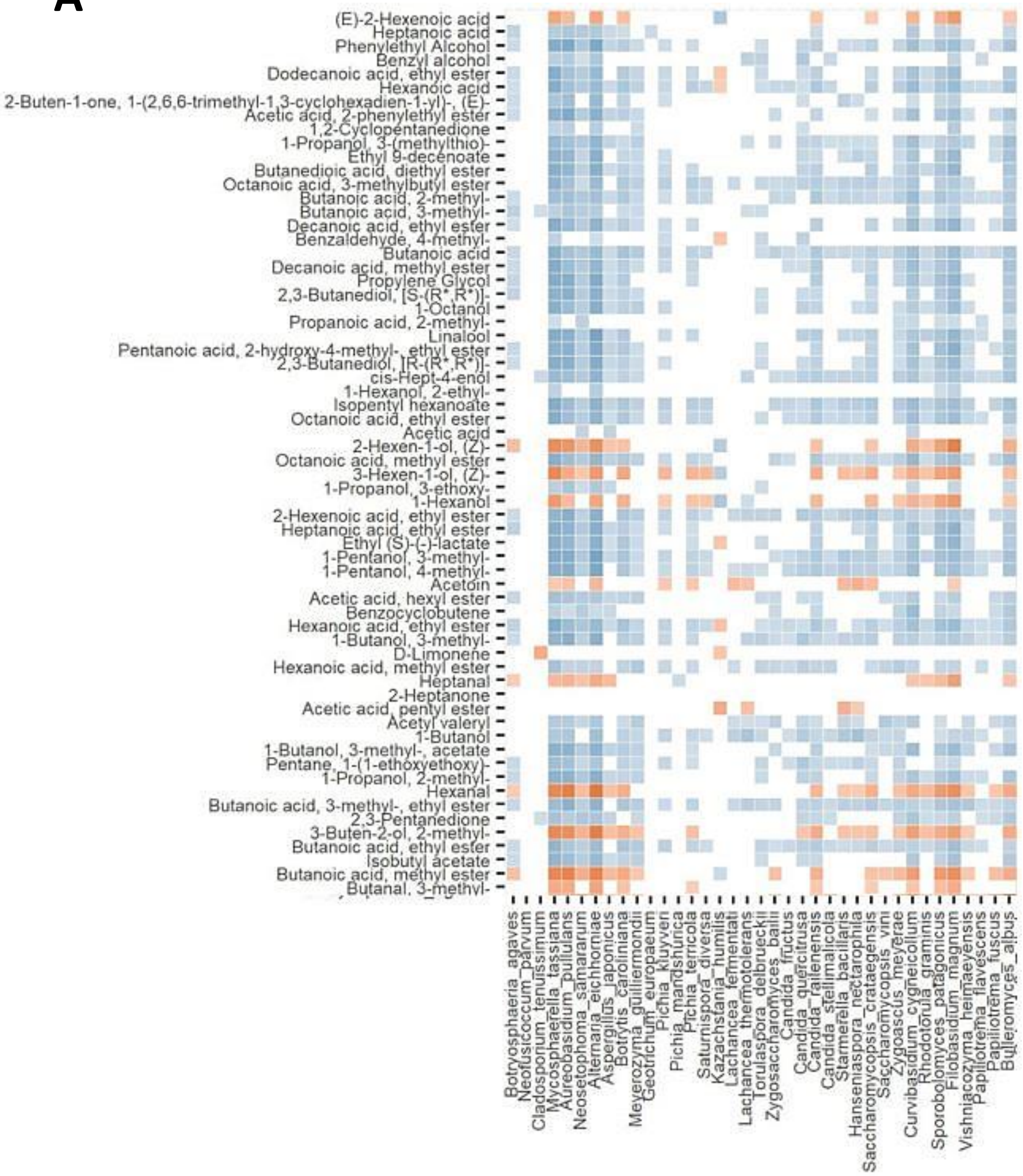

(E)-2-Hexenoic acid
Heptanoic acid
Phenylethyl Alcohol
Benzyl alcohol
Dodecanoic acid, ethyl ester
Hexanoic acid
2-Buten-1-one, 1-(2,6,6-trimethyl-1,3-cyclohexadien-1-yl)-, (E)-
Acetic acid, 2-phenylethyl ester
1,2-Cyclopentanedione
1-Propanol, 3-(methylthio)-
Ethyl 9-decenoate
Butanedioic acid, diethyl ester
Octanoic acid, 3-methylbutyl ester
Butanoic acid, 2-methyl-
Butanoic acid, 3-methyl-
Decanoic acid, ethyl ester
Benzaldehyde, 4-methyl-
Butanoic acid
Decanoic acid, methyl ester
Propylene Glycol
2,3-Butanediol, [S-(R*,R*)]-
1-Octanol
Propanoic acid, 2-methyl-
Linalool
Pentanoic acid, 2-hydroxy-4-methyl-, ethyl ester
2,3-Butanediol, [R-(R*,R*)]-
cis-Hept-4-enol
1-Hexanol, 2-ethyl-
Isopentyl hexanoate
Octanoic acid, ethyl ester
Acetic acid
2-Hexen-1-ol, (Z)-
Octanoic acid, methyl ester
3-Hexen-1-ol, (Z)-
1-Propanol, 3-ethoxy-
1-Hexanol
2-Hexenoic acid, ethyl ester
Heptanoic acid, ethyl ester
Ethyl (S)-(-)-lactate
1-Pentanol, 3-methyl-
1-Pentanol, 4-methyl-
Acetoin
Acetic acid, hexyl ester
Benzocyclobutene
Hexanoic acid, ethyl ester
1-Butanol, 3-methyl-
D-Limonene
Hexanoic acid, methyl ester
Heptanal
2-Heptanone
Acetic acid, pentyl ester
Acetyl valeryl
1-Butanol
1-Butanol, 3-methyl-, acetate
Pentane, 1-(1-ethoxyethoxy)-
1-Propanol, 2-methyl-
Hexanal
Butanoic acid, 3-methyl-, ethyl ester
2,3-Pentanedione
3-Buten-2-ol, 2-methyl-
Butanoic acid, ethyl ester
Isobutyl acetate
Butanoic acid, methyl ester
Butanal, 3-methyl-
Botryosphaeria_agaves
Neofusicoccum_parvum
Cladosporium_tenuissimum
Mycosphaerella_tassiana
Aureobasidium_pullulans
Neosetophoma_samararum
Alternaria_eichhorniae
Aspergillus_japonicus
Botrytis_caroliniana
Meyerozyma_guilliermondii
Geotrichum_europaeum
Pichia_kluyveri
Pichia_mandshurica
Pichia_terricola
Saturnispora_diversa
Kazachstania_humilis
Lachancea_fermentati
Lachancea_thermotolerans
Torulaspora_delbrueckii
Zygosaccharomyces_bailii
Candida_fructus
Candida_quercitrusa
Candida_railenensis
Candida_stellimalicola
Starmerella_bacillaris
Hanseniaspora_nectarophila
Saccharomycopsis_crataegensis
Saccharomycopsis_vini
Zygoascus_meyerae
Curvibasidium_cygneicollum
Rhodotorula_graminis
Sporobolomyces_patagonicus
Filobasidium_magnum
Vishniacozyma_heimaeyensis
Papiliotrema_flavescens
Papiliotrema_fuscus
Bulleromyces_albus

B

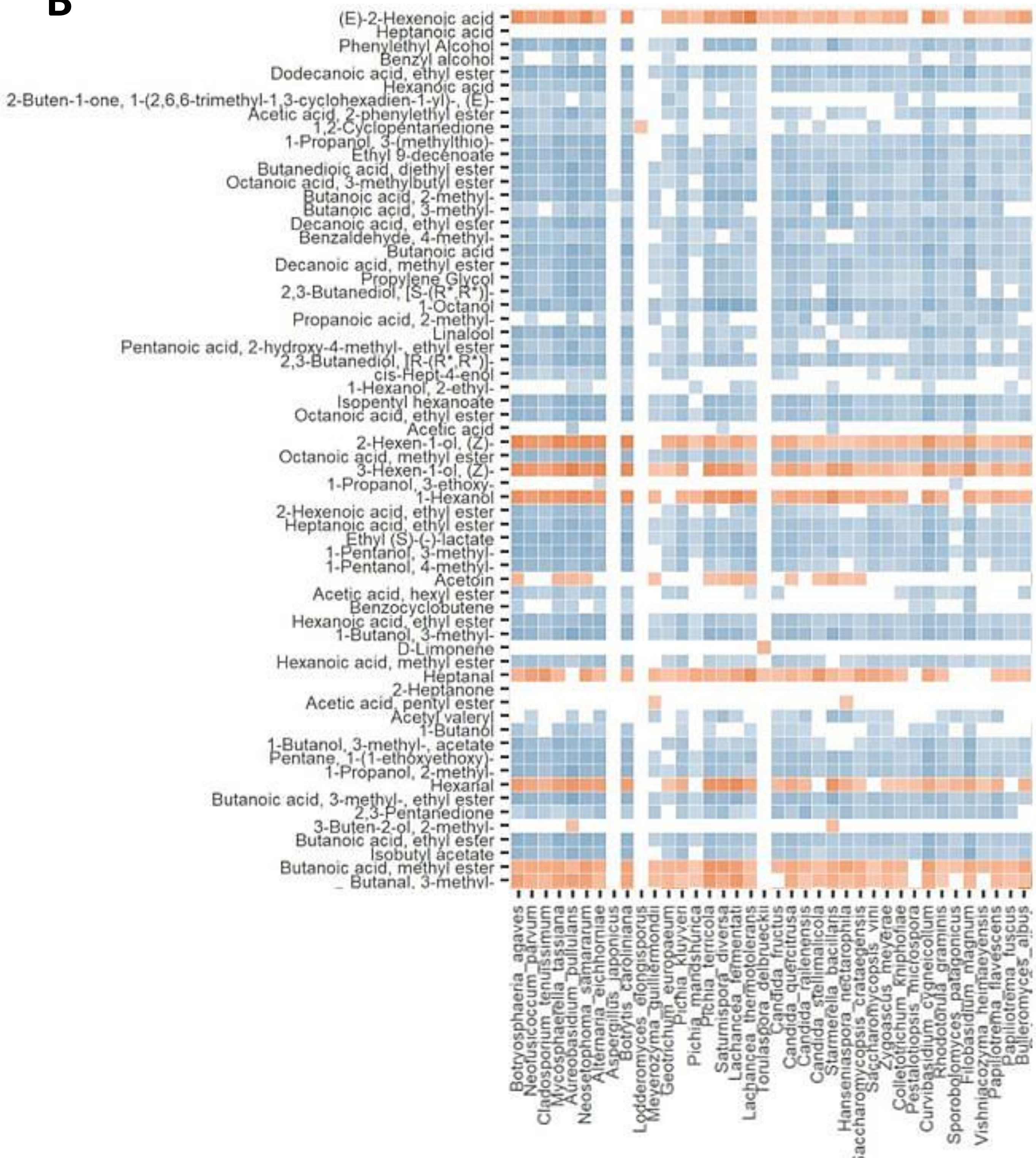
(E)-2-Hexenoic acid
Heptanoic acid
Phenylethyl Alcohol
Benzyl alcohol
Dodecanoic acid, ethyl ester
Hexanoic acid
2-Buten-1-one, 1-(2,6,6-trimethyl-1,3-cyclohexadien-1-yl)-, (E)-
Acetic acid, 2-phenylethyl ester
1,2-Cyclopentanedione
1-Propanol, 3-(methylthio)-
Ethyl 9-decenoate
Butanedioic acid, diethyl ester
Octanoic acid, 3-methylbutyl ester
Butanoic acid, 2-methyl-
Butanoic acid, 3-methyl-
Decanoic acid, ethyl ester
Benzaldehyde, 4-methyl-
Butanoic acid
Decanoic acid, methyl ester
Propylene Glycol
2,3-Butanediol, [S-(R*,R*)]-
1-Octanol
Propanoic acid, 2-methyl-
Linalool
Pentanoic acid, 2-hydroxy-4-methyl-, ethyl ester
2,3-Butanediol, [R-(R*,R*)]-
cis-Hept-4-enol
1-Hexanol, 2-ethyl-
Isopentyl hexanoate
Octanoic acid, ethyl ester
Acetic acid
2-Hexen-1-ol, (Z)-
Octanoic acid, methyl ester
3-Hexen-1-ol, (Z)-
1-Propanol, 3-ethoxy-
1-Hexanol
2-Hexenoic acid, ethyl ester
Heptanoic acid, ethyl ester
Ethyl (S)-(-)-lactate
1-Pentanol, 3-methyl-
1-Pentanol, 4-methyl-
Acetoin
Acetic acid, hexyl ester
Benzocyclobutene
Hexanoic acid, ethyl ester
1-Butanol, 3-methyl-
D-Limonene
Hexanoic acid, methyl ester
Heptanal
2-Heptanone
Acetic acid, pentyl ester
Acetyl valeryl
1-Butanol
1-Butanol, 3-methyl-, acetate
Pentane, 1-(1-ethoxyethoxy)-
1-Propanol, 2-methyl-
Hexanal
Butanoic acid, 3-methyl-, ethyl ester
2,3-Pentanedione
3-Buten-2-ol, 2-methyl-
Butanoic acid, ethyl ester
Isobutyl acetate
Butanoic acid, methyl ester
Butanal, 3-methyl-
Botryosphaeria_agaves
Neofusicoccum_parvum
Cladosporium_tenuissimum
Mycosphaerella_tassiana
Aureobasidium_pullulans
Neosetophoma_samararum
Alternaria_eichhorniae
Aspergillus_japonicus
Botrytis_caroliniana
Lodderomyces_elongisporus
Meyerozyma_guilliermondii
Geotrichum_europaeum
Pichia_kluyveri
Pichia_mandshurica
Pichia_terricola
Saturnispora_diversa
Lachancea_fermentati
Lachancea_thermotolerans
Torulaspora_delbrueckii
Candida_fructus
Candida_quercitrusa
Candida_railenensis
Candida_stellimalicola
Starmerella_bacillaris
Hanseniaspora_nectarophila
Saccharomycopsis_crataegensis
Saccharomycopsis_vini
Zygoascus_meyerae
Colletotrichum_kniphofiae
Pestalotiopsis_microspora
Curvibasidium_cygneicollum
Rhodotorula_graminis
Sporobolomyces_patagonicus
Filobasidium_magnum
Vishniacozyma_heimaeyensis
Papiliotrema_flavescens
Papiliotrema_fuscus
Bulleromyces_albus

C

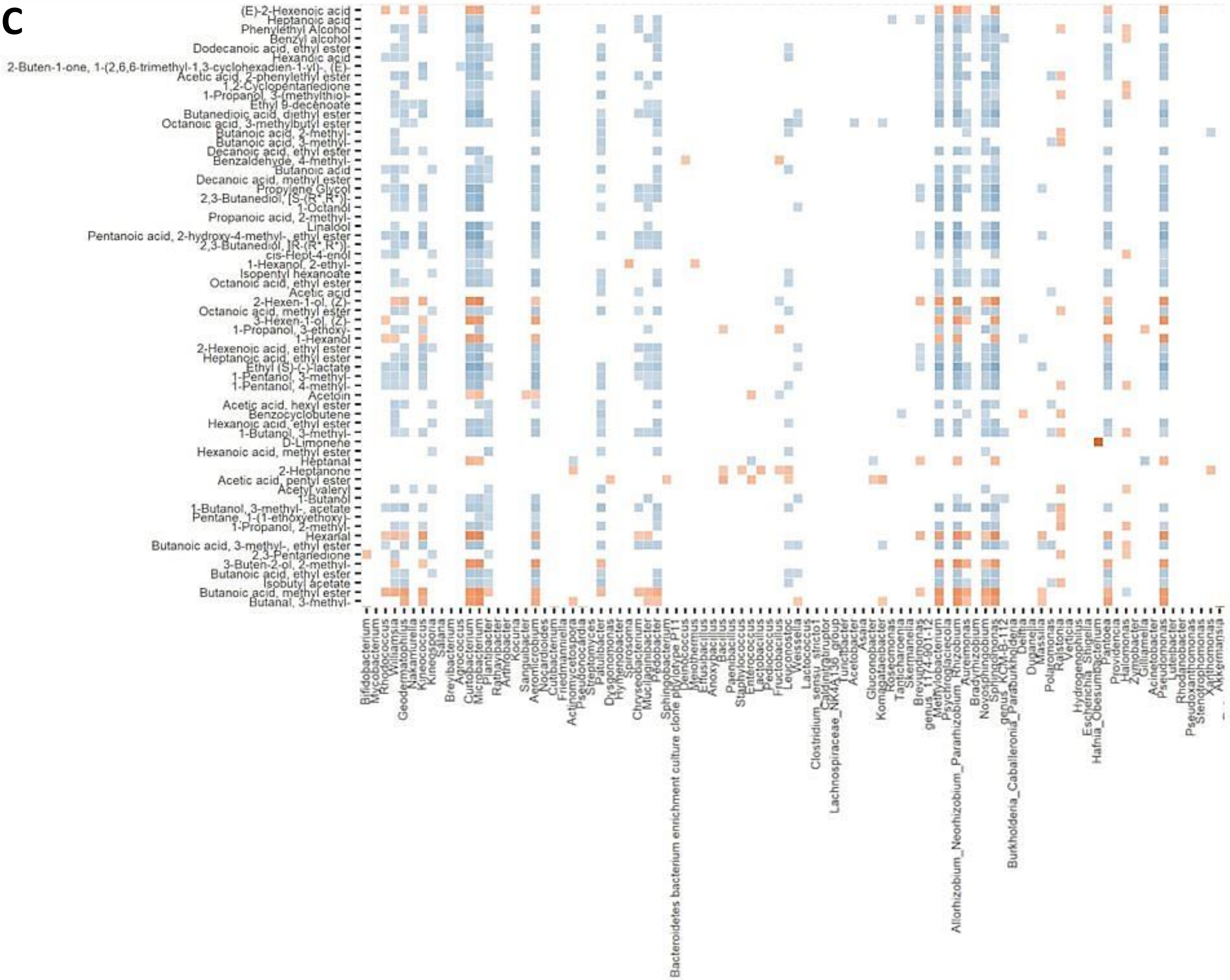
(E)-2-Hexenoic acid
Heptanoic acid
Phenylethyl Alcohol
Benzyl alcohol
Dodecanoic acid, ethyl ester
Hexanoic acid
2-Buten-1-one, 1-(2,6,6-trimethyl-1,3-cyclohexadien-1-yl)-, (E)-
Acetic acid, 2-phenylethyl ester
1,2-Cyclopentanedione
1-Propanol, 3-(methylthio)-
Ethyl 9-decenoate
Butanedioic acid, diethyl ester
Octanoic acid, 3-methylbutyl ester
Butanoic acid, 2-methyl-
Butanoic acid, 3-methyl-
Decanoic acid, ethyl ester
Benzaldehyde, 4-methyl-
Butanoic acid
Decanoic acid, methyl ester
Propylene Glycol
2,3-Butanediol, [S-(R*,R*)]-
1-Octanol
Propanoic acid, 2-methyl-
Linalool
Pentanoic acid, 2-hydroxy-4-methyl-, ethyl ester
2,3-Butanediol, [R-(R*,R*)]-
cis-Hept-4-enol
1-Hexanol, 2-ethyl-
Isopentyl hexanoate
Octanoic acid, ethyl ester
Acetic acid
2-Hexen-1-ol, (Z)-
Octanoic acid, methyl ester
3-Hexen-1-ol, (Z)-
1-Propanol, 3-ethoxy-
1-Hexanol
2-Hexenoic acid, ethyl ester
Heptanoic acid, ethyl ester
Ethyl (S)-(-)-lactate
1-Pentanol, 3-methyl-
1-Pentanol, 4-methyl-
Acetoin
Acetic acid, hexyl ester
Benzocyclobutene
Hexanoic acid, ethyl ester
1-Butanol, 3-methyl-
D-Limonene
Hexanoic acid, methyl ester
Heptanal
2-Heptanone
Acetic acid, pentyl ester
Acetyl valeryl
1-Butanol
1-Butanol, 3-methyl-, acetate
Pentane, 1-(1-ethoxyethoxy)-
1-Propanol, 2-methyl-
Hexanal
Butanoic acid, 3-methyl-, ethyl ester
2,3-Pentanedione
3-Buten-2-ol, 2-methyl-
Butanoic acid, ethyl ester
Isobutyl acetate
Butanoic acid, methyl ester
Butanal, 3-methyl-
Bifidobacterium
Mycobacterium
Rhodococcus
Williamsia
Geodermatophilus
Nakamurella
Kineococcus
Kineosporia
Salana
Brevibacterium
Agrococcus
Curtobacterium
Microbacterium
Plantibacter
Rathayibacter
Arthrobacter
Kocuria
Sanguibacter
Aeromicrobium
Nocardioides
Cutibacterium
Friedmanniella
Actinomycetospora
Pseudonocardia
Streptomyces
Patulibacter
Dyadobacter
Hymenobacter
Spirosoma
Chryseobacterium
Mucilaginibacter
Pedobacter
Sphingobacterium
Bacteroidetes bacterium enrichment culture clone phylotype P11
Deinococcus
Meiothermus
Effusibacillus
Anoxybacillus
Bacillus
Paenibacillus
Staphylococcus
Enterococcus
Lactobacillus
Pediococcus
Fructobacillus
Leuconostoc
Weissella
Lactococcus
Clostridium_sensu_stricto1
Caldicellulosiruptor
Lachnospiraceae_NK4A136_group
Tunicibacter
Acetobacter
Asaia
Gluconobacter
Komagataeibacter
Roseomonas
Tanticharoenia
Skermanella
Brevundimonas
genus_1174-901-12
Methylobacterium
Psychroglaciecola
Allorhizobium_Neorhizobium_Pararhizobium_Rhizobium
Aureimonas
Bradyrhizobium
Novosphingobium
Sphingomonas
genus_KCM-B-112
Burkholderia_Caballeronia_Paraburkholderia
Delftia
Duganella
Massilia
Polaromonas
Ralstonia
Variovorax
Hydrogenophilus
Escherichia_Shigella
Hafnia_Obesumbacterium
Pantoea
Providencia
Halomonas
Zymobacter
Gilliamella
Acinetobacter
Pseudomonas
Luteibacter
Rhodanobacter
Pseudoxanthomonas
Stenotrophomonas
Xanthomonas
Akkermansia

D

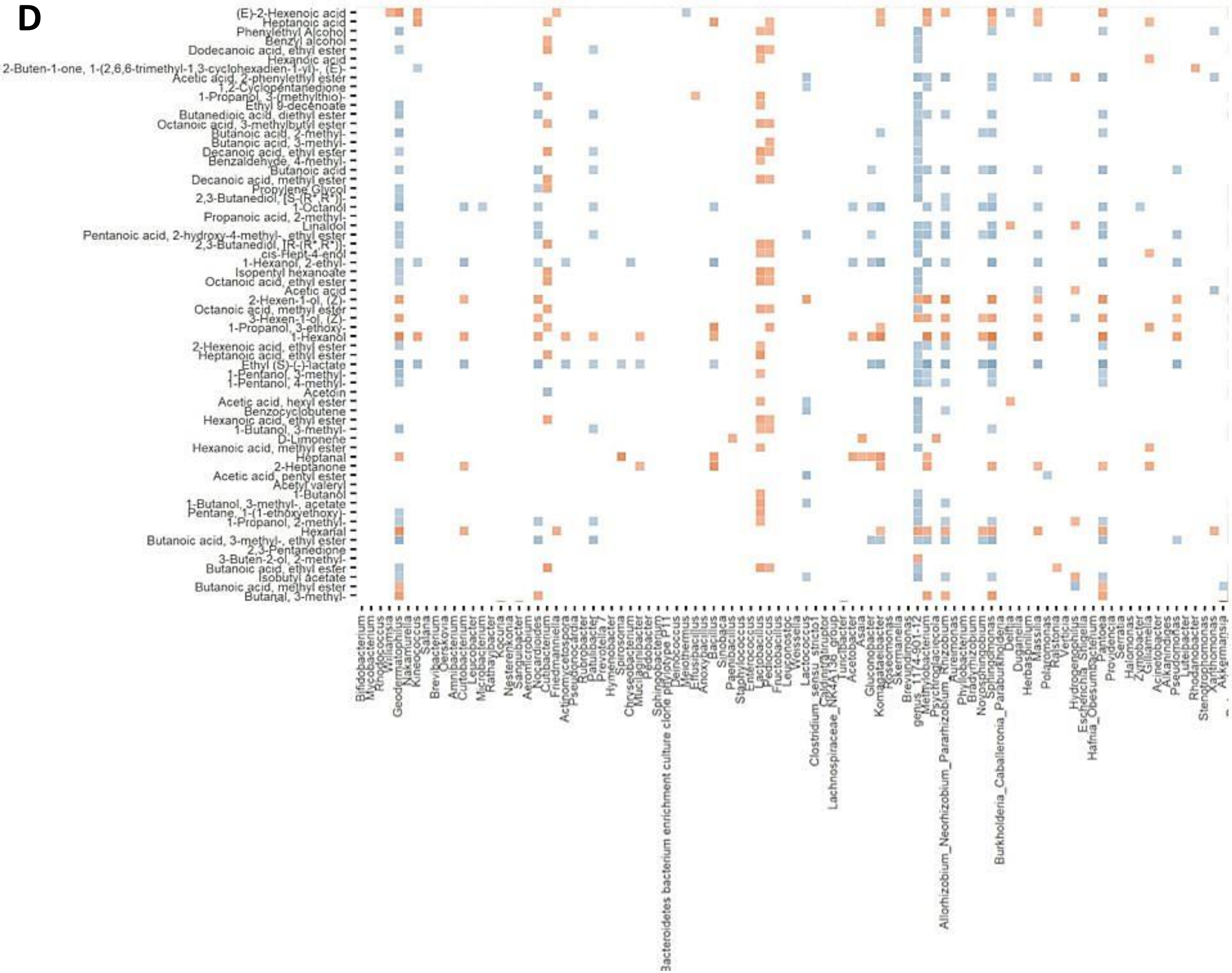

(E)-2-Hexenoic acid
Heptanoic acid
Phenylethyl Alcohol
Benzyl alcohol
Dodecanoic acid, ethyl ester
Hexanoic acid
2-Buten-1-one, 1-(2,6,6-trimethyl-1,3-cyclohexadien-1-yl)-, (E)-
Acetic acid, 2-phenylethyl ester
1,2-Cyclopentanedione
1-Propanol, 3-(methylthio)-
Ethyl 9-decenoate
Butanedioic acid, diethyl ester
Octanoic acid, 3-methylbutyl ester
Butanoic acid, 2-methyl-
Butanoic acid, 3-methyl-
Decanoic acid, ethyl ester
Benzaldehyde, 4-methyl-
Butanoic acid
Decanoic acid, methyl ester
Propylene Glycol
2,3-Butanediol, [S-(R*,R*)]-
1-Octanol
Propanoic acid, 2-methyl-
Linalool
Pentanoic acid, 2-hydroxy-4-methyl-, ethyl ester
2,3-Butanediol, [R-(R*,R*)]-
cis-Hept-4-enol
1-Hexanol, 2-ethyl-
Isopentyl hexanoate
Octanoic acid, ethyl ester
Acetic acid
2-Hexen-1-ol, (Z)-
Octanoic acid, methyl ester
3-Hexen-1-ol, (Z)-
1-Propanol, 3-ethoxy-
1-Hexanol
2-Hexenoic acid, ethyl ester
Heptanoic acid, ethyl ester
Ethyl (S)-(-)-lactate
1-Pentanol, 3-methyl-
1-Pentanol, 4-methyl-
Acetoin
Acetic acid, hexyl ester
Benzocyclobutene
Hexanoic acid, ethyl ester
1-Butanol, 3-methyl-
D-Limonene
Hexanoic acid, methyl ester
Heptanal
2-Heptanone
Acetic acid, pentyl ester
Acetyl valeryl
1-Butanol
1-Butanol, 3-methyl-, acetate
Pentane, 1-(1-ethoxyethoxy)-
1-Propanol, 2-methyl-
Hexanal
Butanoic acid, 3-methyl-, ethyl ester
2,3-Pentanedione
3-Buten-2-ol, 2-methyl-
Butanoic acid, ethyl ester
Isobutyl acetate
Butanoic acid, methyl ester
Butanal, 3-methyl-
Bifidobacterium
Mycobacterium
Rhodococcus
Williamsia
Geodermatophilus
Nakamurella
Kineococcus
Salana
Brevibacterium
Oerskovia
Amnibacterium
Curtobacterium
Leucobacter
Microbacterium
Rathayibacter
Kocuria
Nesterenkonia
Sanguibacter
Aeromicrobium
Nocardioides
Cutibacterium
Friedmanniella
Actinomycetospora
Pseudonocardia
Rubrobacter
Patulibacter
Prevotella 7
Hymenobacter
Spirosoma
Chryseobacterium
Mucilaginibacter
Pedobacter
Sphingobacterium
Bacteroidetes bacterium enrichment culture clone phylotype P11
Deinococcus
Meiothermus
Effusibacillus
Anoxybacillus
Bacillus
Sinobaca
Paenibacillus
Staphylococcus
Enterococcus
Lactobacillus
Pediococcus
Fructobacillus
Leuconostoc
Weissella
Lactococcus
Clostridium_sensu_stricto1
Lachnospiraceae_NK4A136_group
Turicibacter
Acetobacter
Asaia
Gluconobacter
Komagataeibacter
Roseomonas
Skermanella
Brevundimonas
genus_1174-901-12
Methylobacterium
Psychroglaciecola
Allorhizobium_Neorhizobium_Pararhizobium_Rhizobium
Aureimonas
Phyllobacterium
Bradyrhizobium
Novosphingobium
Sphingomonas
Burkholderia_Caballeronia_Paraburkholderia
Delftia
Duganella
Herbaspirillum
Massilia
Polaromonas
Ralstonia
Hydrogenophilus
Escherichia_Shigella
Hafnia_Obesumbacterium
Pantoea
Providencia
Serratia
Halomonas
Zymobacter
Gilliamella
Acinetobacter
Alkanindiges
Pseudomonas
Luteibacter
Rhodanobacter
Stenotrophomonas
Xanthomonas
Akkermansia

Supplementary Figure 4. Spearman's correlation analysis between microbial communities and volatile metabolites identified across fermentation stages. Data shown here are the correlation coefficients between (A and B) fungi (Species level) or (C and D) bacteria (Genus level) and volatile metabolites in the (A and C) Central and the (B and D) East regions visualized in the heatmaps showing positive correlation (orange) and negative correlation (grey). Spearman's correlation significance indicated by coefficient with q-value < 0.05 (coefficients with q > 0.05 are shown in white block).
